# Supplementary material for: Conformational changes in Lassa virus L protein associated with promoter binding and RNA synthesis activity
Source: Nat Commun. 2021 Dec 2;12:7018. doi: 10.1038/s41467-021-27305-5 (PMC8639829; doi:10.1038/s41467-021-27305-5)
Supplement: Supplementary file 1 — Supplementary Information [file 41467_2021_27305_MOESM1_ESM.pdf]

Supplementary tables and figures

Conformational changes in Lassa virus L protein associated with promoter binding and RNA synthesis activity

Tomas Kouba[1], Dominik Vogel[2], Sigurdur R. Thorkelsson[3], Emmanuelle R. J. Quemin[3], Harry M. Williams[2], Morlin Milewski[2], Carola Busch[2], Stephan Günther[2], Kay Grünewald[3], Maria Rosenthal[2,*] and Stephen Cusack[1,*]

1- European Molecular Biology Laboratory, Grenoble, France

2- Bernhard Nocht Institute for Tropical Medicine, Hamburg, Germany

3- Centre for Structural Systems Biology, Leibniz Institute for Experimental Virology, University of Hamburg, Hamburg, Germany

These authors contributed equally: Tomas Kouba, Dominik Vogel, Sigurdur R. Thorkelsson

These authors jointly supervised this work: Maria Rosenthal and Stephen Cusack

* To whom correspondence should be addressed: rosenthal@bnitm.de and cusack@embl.fr

Supplementary Table 1: Synthetic RNA oligos used in the assays. The table lists the RNA oligonucleotides that were used in the described assays. The specific sequence, length in nucleotides and the identifier used to label the RNA in the experimental descriptions are given. Underlined nucleotides in the 5' (nts 0-19) and 3' (nts 1-19) indicate mismatches between the two otherwise complementary RNA strands. All RNAs were synthesized by Biomers, TriLink BioTechnologies or Chemgenes.

	Length [nt]	Identifier		Sequence	
vRNA	20	5' (0-19)	5' HO-	GCGCACCGGGGAUCCUAGGC	-OH 3'
	20	5' (0-19) Pi	5' HO-	GCGCACCGGGGAUCCUAGGC	-PO_4 3'
	10	5' (10-19)	5' HO-	GAUCCUAGGC	-OH 3'
	19	3' (1-19)	3' HO-	GCGUGUCACCUAGGAUCCG	-OH 5'
	19	3' (1-19) Pi	3' PO_4-	GCGUGUCACCUAGGAUCCG	-OH 5'
	16	3' (1-16)	3' HO-	GCGUGUCACCUAGGAU	
EMSA	10	3' (1-10)	3' HO-	GCGUGUCACC	-Cy3 5'
EN	17	PPP-RNA	5' ppp-	AAACGCAACAACAACAC	-Cy5 3'
	18	Cap-RNA	5' Cap^0-	AAACGCAAGCAACAACAC	-Cy5 3'
Primer	6	St1	5' HO-	GCGCAC	-OH 3'
	6	HO-C1	5' HO-	AAACGC	-OH 3'
	6	ppp-C1	5' ppp-	AAACGC	-OH 3'
	6	Cap^0-C1	5' Cap^0-	AAACGC	-OH 3'
	6	Cap^1-C1	5' Cap^0-	A_mAACGC	-OH 3'
	10	C8	5' HO-	AAUAAUACGC	-OH 3'
	3	GCG	5' HO-	GCG	-OH 3'

Supplementary Table 2. Cryo-EM data collection, refinement and validation statistics.

This table provides the statistics for the data collection, refinement and structure validation of the cryo-EM structures. Refinement statistics were generated using Phenix comprehensive validation software.

	APO-CORE	APO-ENDO	APO-RIBBON	3'END-CORE	3'END-ENDO	PRE-INITIATION	DISTAL-PROMOTER	MID-LINK	ELONGATION
PDB	**7OCH**	**7OE3**	**7OE7**	**7OEA**	**7OEB**	**7OJL**	**7OJJ**	**7OJK**	**7OJN**
EMDB	**EMD-12807**	**EMD-12860**	**EMD-12861**	**EMD-12862**	**EMD-12863**	**EMD-12955**	**EMD-12953**	**EMD-12954**	**EMD-12956**
**Data collection and processing**	CSSB DATA 1			ESRF DATA		CSSB DATA 3	CSSB DATA 2		CSSB DATA 4
Microscope	CSSB FEI Titan Krios			ESRF FEI Titan Krios		CSSB FEI Titan Krios	CSSB FEI Titan Krios		CSSB FEI Titan Krios
Voltage (kV)	300			300		300	300		300
Camera	Gatan K3 BioQuantum			Gatan K2 Quantum		Gatan K3 BioQuantum	Gatan K3 BioQuantum		Gatan K3 BioQuantum
Magnification	105,000			165,000		105,000	105,000		105,000
Nominal negative defocus range (µm)	0.4 - 3.1			0.3 - 2.8		0.5 - 2.0	0.4 - 3.1		0.6 - 2.6
Exposure time (s)	2.5			5.06		2.5	2.5		2.5
Electron exposure (e–/Å^2)	49.5			46		53	49.5		51.7
Number of frames collected (no.)	50			46		50	50		50
Number of frames processed (no.)	23			26		50	25		50
Pixel size (Å)	0.87			0.827		0.85	0.87		0.85
Micrographs (no.)	15,448			6,616		13,204	13,462		10,368
Total particle images (no.)	2,651,000			1,468,000		2,469,716	2,882,000		2,452,281
**Refinement**									
Particles per class (no.)	67.5 k	74 k	35.4 k	84.5 k	40.2 k	119.9 k	23.7 k	40.7 k	79.6 k
Map resolution (Å), 0.143 FSC	3.14	3.35	3.73	2.70	3.04	3.34	3.89	3.50	2.92
Map sharpening *B* factor (Å^2)	-80.2	-87.1	-93.5	-42.8	-56.7	-104.1	-124.6	-104.3	-45.9
Map versus model cross-correlation CC-MASK	0.8337	0.8783	0.8089	0.8374	0.8114	0.8387	0.8183	0.8804	0.8576
**Model composition**									
Non-hydrogen atoms	9516	11904	11116	11703	11682	12842	15316	14330	16991
Protein residues	1181	1478	1382	1432	1434	1498	1843	1785	2010
Nucleotide residues	0	0	0	7	6	33	21	0	39
Water	0	0	0	17	1	0	0	0	0
Ligands	1 Mg 1 Zn	1 Mg 1 Zn	1 Mg 1 Zn	1 Mg 1 Zn	2 Mg 1 Zn	1 Mn	1 Zn	1 Mg 1 Zn	5 Mn, 1 Zn UMPNPP

***Mean B* factors ($Å^2$)**									
Protein	54.51	119.18	68.49	69.63	44.51	33.48	157.22	124.91	41.45
Nucleotide	-	-	-	50.71	37.82	48.20	144.01	-	46.47
Ligand	75.78	115.19	78.81	70.43	64.00	30.71	178.60	137.47	32.66
Water	-	-	-	30.48	17.56	-	-	-	-
**R.m.s. deviations**									
Bond lengths (Å)	0.003	0.003	0.002	0.004	0.004	0.002	0.002	0.006	0.002
Bond angles (°)	0.537	0.519	0.528	0.772	0.557	0.456	0.614	0.663	0.405
**Validation**									
MolProbity score	2.05	2.22	1.60	1.66	1.71	2.25	2.19	2.08	1.47
All-atom clashscore	3.89	5.91	3.72	6.04	7.67	7.20	10.05	9.33	3.51
Poor rotamers (%)	3.8	3.39	0.0	0.08	0.53	0.30	0.0	0.12	0.93
**Ramachandran plot**									
Favored (%)	93.44	91.84	93.23	95.31	95.74	91.78	84.59	88.64	95.24
Allowed (%)	6.56	8.16	6.77	4.47	4.04	8.15	15.19	11.36	4.71
Outliers (%)	0	0	0	0.21	0.21	0.0	0.22	0	0.05

Supplementary Table 3: Overview of residues included in each model. For each structure of LASV L protein, the residue ranges defined within the respective map are listed.

Structure	Residues Ranges
APO-CORE	199 – 336, 344 – 405, 410 – 472, 477 – 514, 533 – 803, 1102 – 1214, 1219 – 1258, 1266 – 1560, 1610 – 1709, 1717 – 1730, 1777 – 1823
APO-ENDO	1 – 403, 410 – 472, 476 – 513, 531 – 804, 1087 – 1259, 1264 – 1559, 1575 – 1730, 1737 – 1762, 1774 – 1800, 1803 – 1824
APO-RIBBON	199 – 308, 311 – 338, 342 – 404, 410 – 513, 531 – 812, 816 – 886, 903 – 925, 1055 – 1560, 1578 – 1607, 1611 – 1730, 1777 – 1800, 1803 – 1823
3END-CORE	2 – 192, 198 – 308, 319 – 339, 341 – 474, 478 – 514, 527 – 802, 1089 – 1212, 1218 – 1259, 1264 – 1559, 1578 – 1730, 1777 – 1801, 1803 – 1824
3END-ENDO	1 – 192, 198 – 308, 319 – 339, 341 – 474, 478 – 514, 527 – 802, 1088 – 1212, 1218 – 1259, 1264 – 1559, 1578 – 1730, 1777 – 1824
MID-LINK	1 – 337, 344 – 405, 410 – 514, 531 – 812, 816 – 885, 896 – 925, 1059 – 1559, 1611 – 1730, 1739 – 1790, 1805 – 1823, 1833 – 1907, 2077 – 2208
DISTAL-PROMOTER	1 – 309, 318 – 405, 410 – 811, 825 – 886, 896 – 925, 939 – 1002, 1013 – 1040, 1055 – 1559, 1611 – 1730, 1739 – 1790, 1804 – 1823, 1834 – 1907, 2077 – 2165
PRE-INITIATION	199 – 307, 319 – 404, 410 – 811, 819 – 884, 902 – 926, 940 – 1002, 1013 – 1039, 1053 – 1087, 1093 – 1566, 1575 – 1589, 1609 – 1730, 1738 – 1763, 1776 – 1823
ELONGATION	1 – 307, 315 – 404, 410 – 863, 870 – 882, 910 – 925, 1059 – 1562, 1577 – 1824, 1831 – 2208

Supplementary Fig. 1

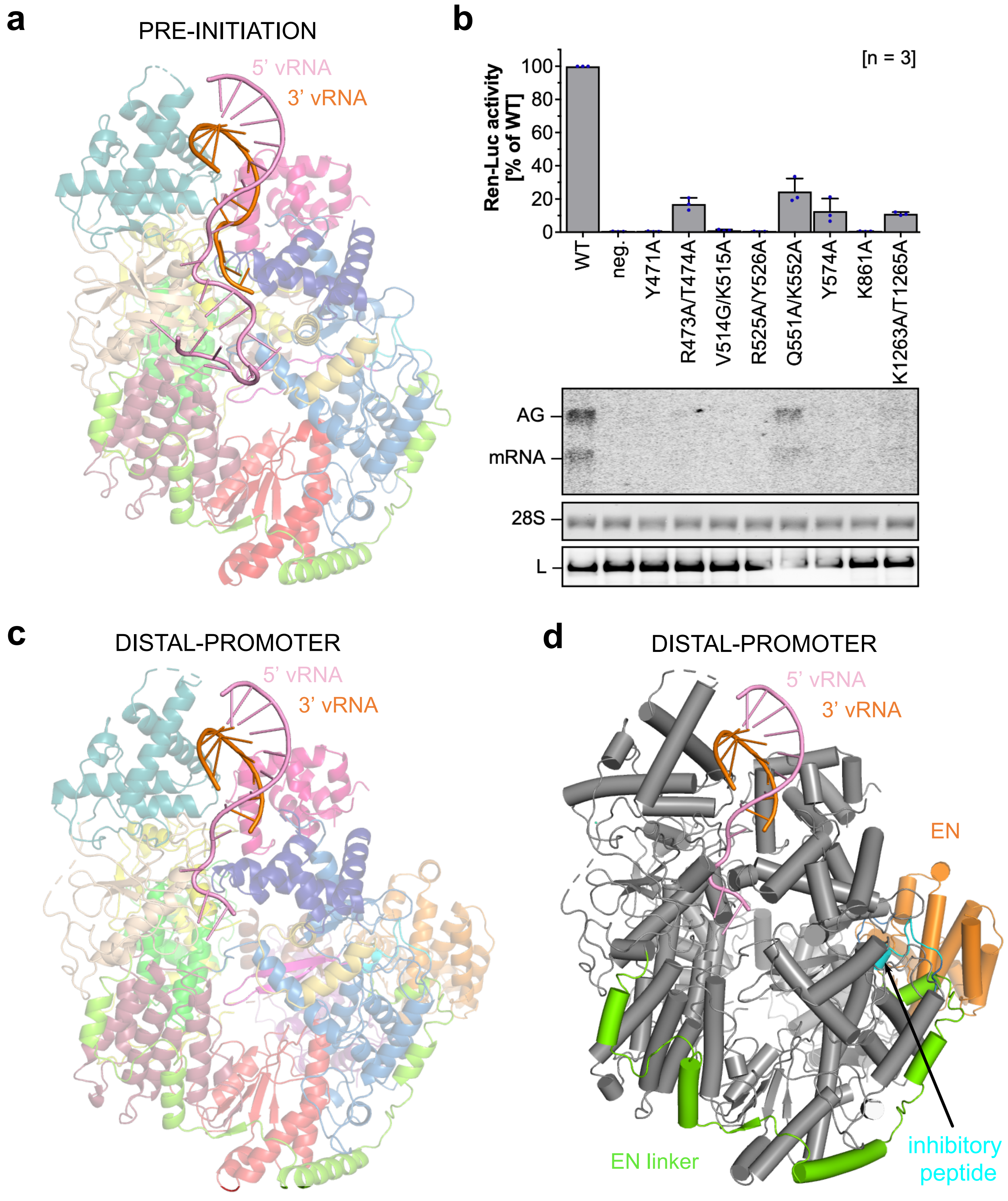

Supplementary Figure 1. PRE-INITIATION and DISTAL-PROMOTER structures. **a,** An overview of the PRE-INITIATION structure is shown with the promoter RNA highlighted in orange (3' vRNA) and pink (5' vRNA). **b,** LASV mini-replicon data for L proteins with mutations in the 5' hook RNA binding site presenting luciferase reporter activity (in standardized relative light units relative to the wild-type L protein (WT)). Data are presented as mean values +/- SD of 3 biological replicates (n=3). All biological replicates are shown as blue dots (top panel). Middle panels present Northern blotting results with signals for antigenomic viral RNA (AG), viral mRNA (mRNA) and 28S ribosomal RNA (28S) as a loading control, and bottom panel shows Western blot detection of FLAG-tagged L proteins (L) to demonstrate general expressibility of the mutants. Source data are provided as a Source Data file. **c,** The DISTAL-PROMOTER structure is shown with the promoter RNA highlighted in orange (3' vRNA) and pink (5' vRNA). **d,** The conformation of the EN domain (orange) and EN linker (green) is highlighted in a ribbon diagram presentation of the DISTAL-PROMOTER structure. Note that the EN is not resolved in the PRE-INITIATION structure.

Supplementary Fig. 2

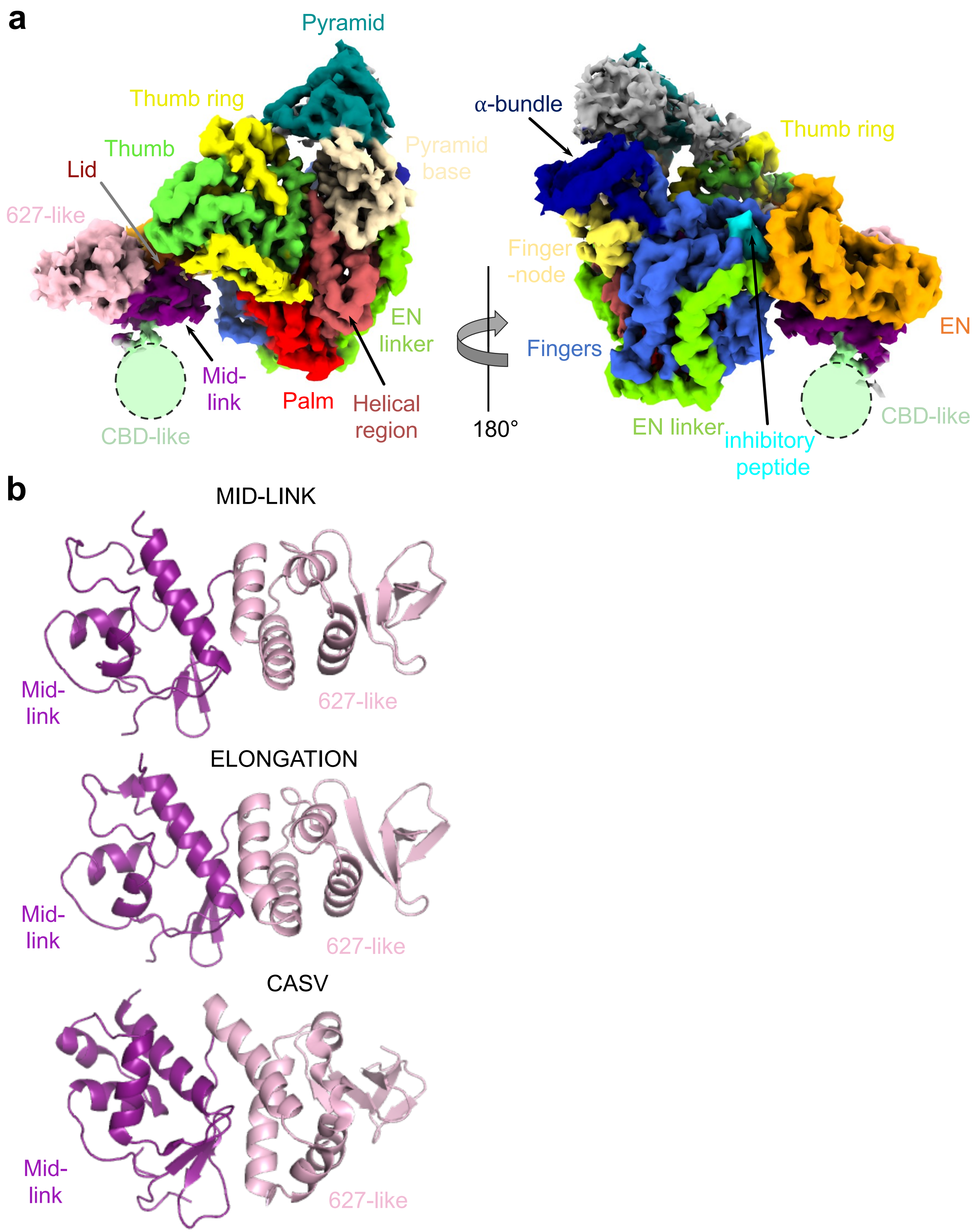

Supplementary Figure 2. MID-LINK structure. a, The cryo-EM map of the MID-LINK structure is shown from two perspectives as indicated. Domains are colored and labelled according to Fig. 3. Additional low-resolution density for the CBD-like domain is indicated by a dashed green circle. **b,** Comparison of only the mid-link (violet) and 627-like (pink) domains of MID-LINK and ELONGATION structures as well as the CASV crystal structure of the L protein C-terminal region (PDB:5MUS).

Supplementary Fig. 3

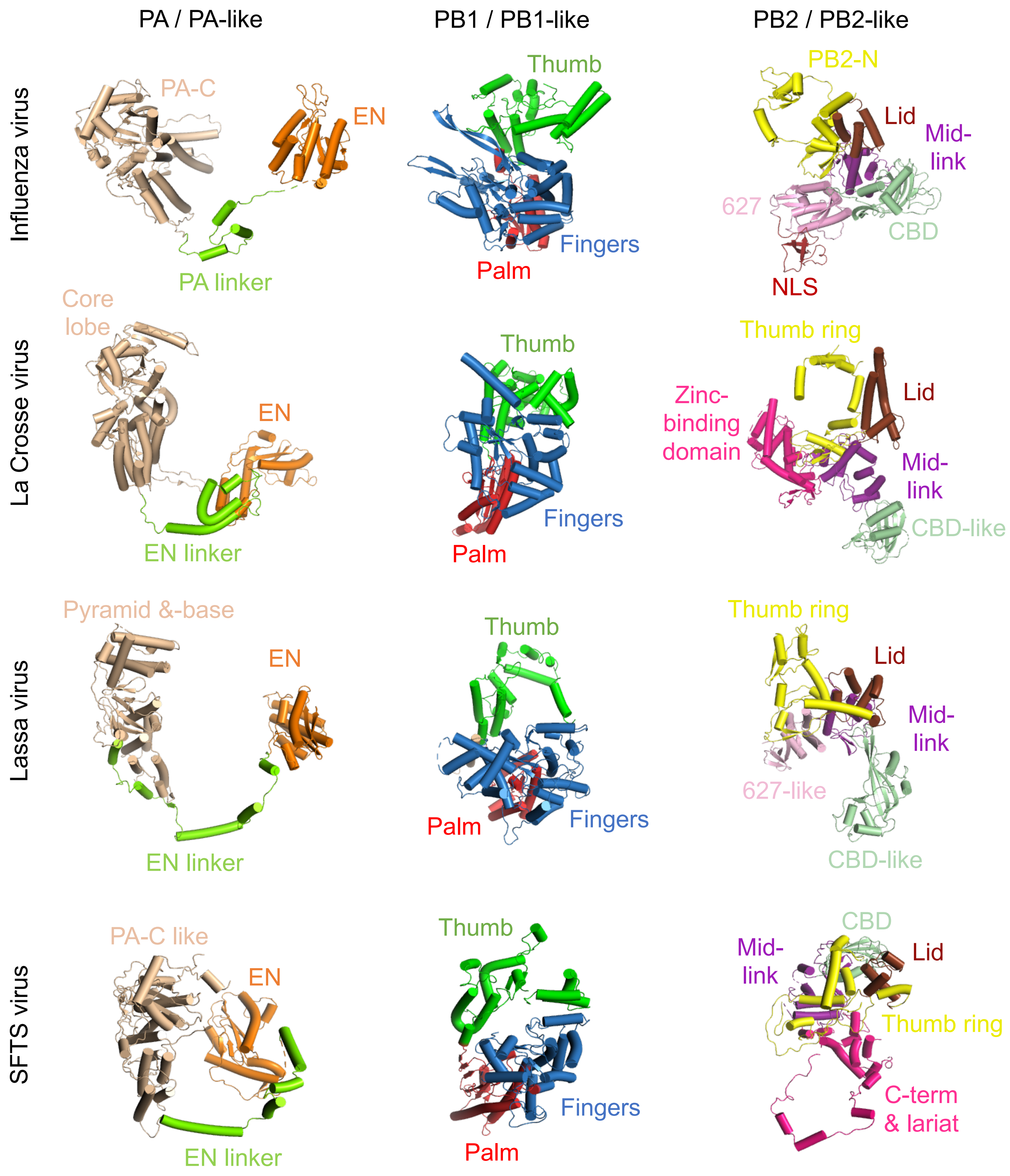

Supplementary Figure 3. Comparison of related polymerase structures. The structures of influenza virus (PDB: 6T0V, elongation state), LACV (PDB: 6Z8K, elongation state), LASV (ELONGATION), and Severe fever with thrombocytopenia syndrome virus (SFTS virus, PDB: 7ALP, apo-form) are shown side by side. Structures were divided according to influenza virus PA, PB1 and PB2 subunits and corresponding elements are shown in the same color.

Supplementary Fig. 4

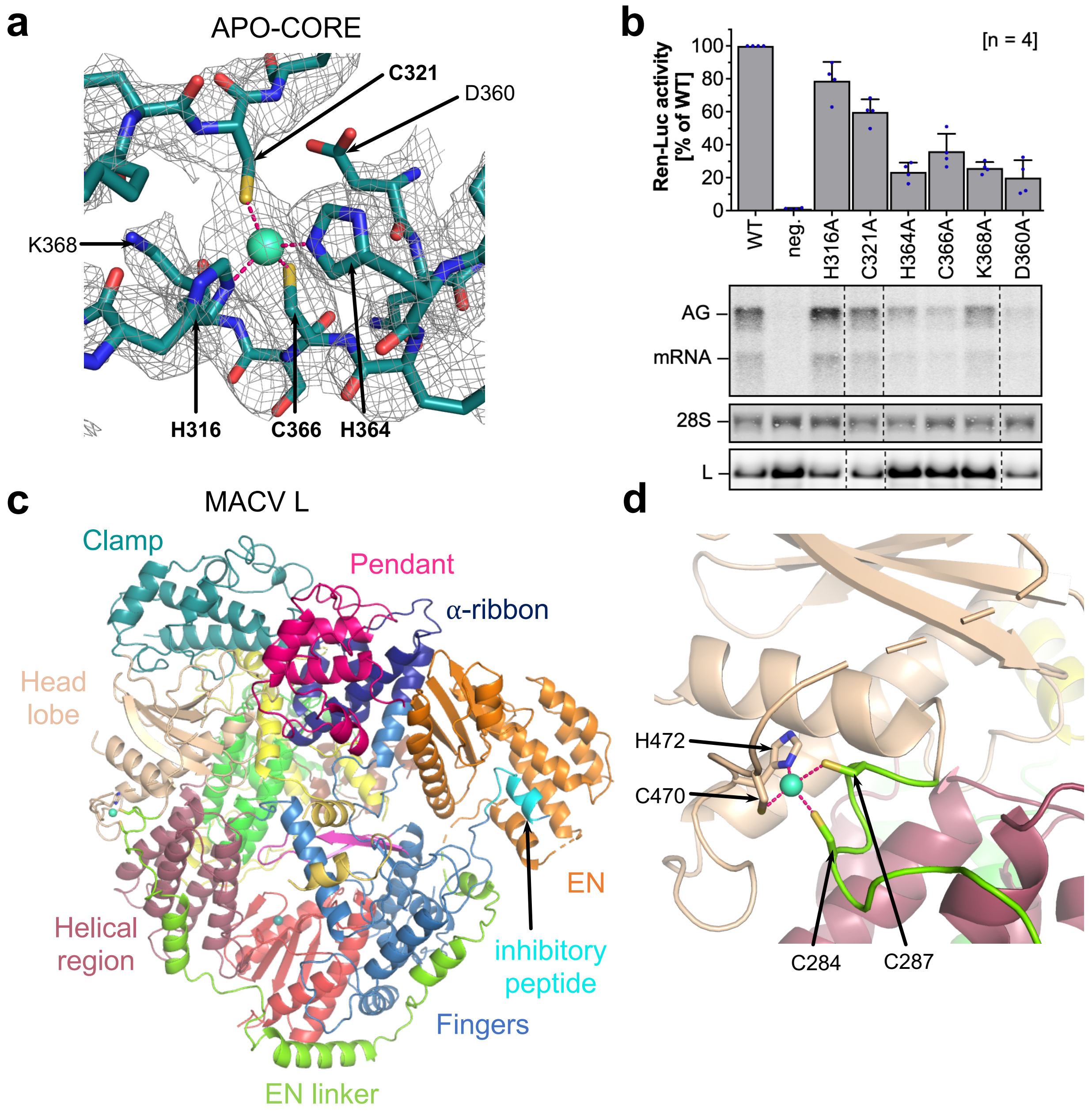

Supplementary Figure 4. Structural Zinc-binding site in the pyramid domain. a, Close-up of the Zinc-binding site of APO-CORE structure with the protein chain shown as sticks (teal) and the Zinc ion as sphere (cyan) together with the experimental map shown as a grey mesh. Co-ordination is indicated by red doted lines. **b,** LASV mini-replicon data for L proteins with mutations in and around the Zinc-binding site presenting luciferase reporter activity (in standardized relative light units relative to the wild-type L protein (WT)). Data are presented as mean values +/- SD of 4 biological replicates (n=4). All biological replicates are shown as blue dots (top panel). Middle panels present Northern blotting results with signals for antigenomic viral RNA (AG), viral mRNA (mRNA) and 28S ribosomal RNA (28S) as a loading control, and bottom panel shows Western blot detection of FLAG-tagged L proteins (L) to demonstrate general expressibility of the mutants. Source data are provided as a Source Data file. **c,** Overview of the L protein of MACV (PDB:6KLE) as a ribbon diagram. Corresponding domains are colored according to Fig. 3 and labelled. **d,** Close-up of the MACV Zinc-binding site. Colors according to (c).

Supplementary Fig. 5

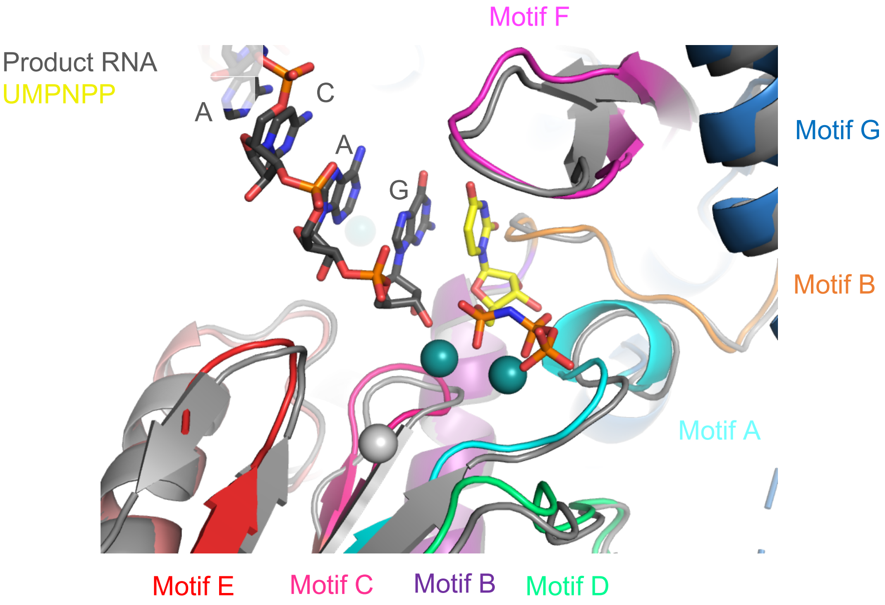

Supplementary Figure 5. Polymerase active site in ELONGATION and APO-CORE structures. Superimposition of the RdRp active sites of the ELONGATION (colored) and APO-CORE (grey) structures as ribbon diagram. Conserved RdRp motifs are shown in distinct colors and with labels. Product RNA (grey) and non-hydrolysable UTP (UMPNPP, yellow) of the ELONGATION structure are shown as sticks.

Supplementary Fig. 6

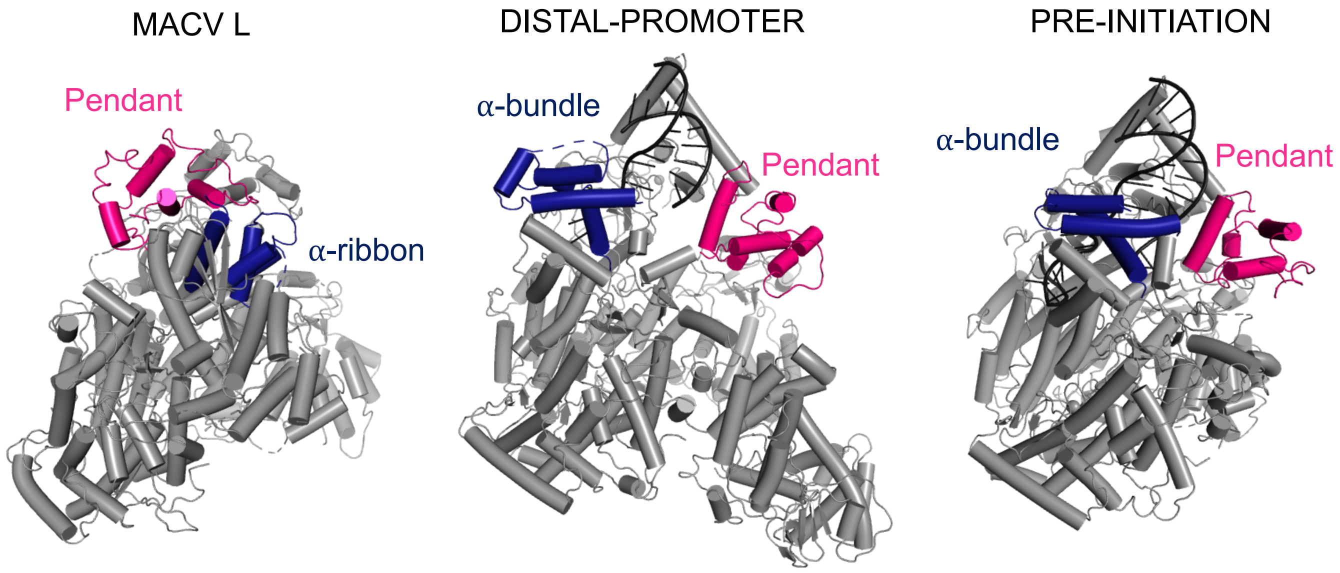

Supplementary Figure 6. Pendant and α-bundle domains. Comparison of MACV L (PDB: 6KLE), LASV DISTAL-PROMOTER and LASV PRE-INITIATION structures as ribbon diagrams with the pendant (pink) and α-bundle/ribbon (dark blue) domains shown in color and indicated by name.

Supplementary Fig. 7

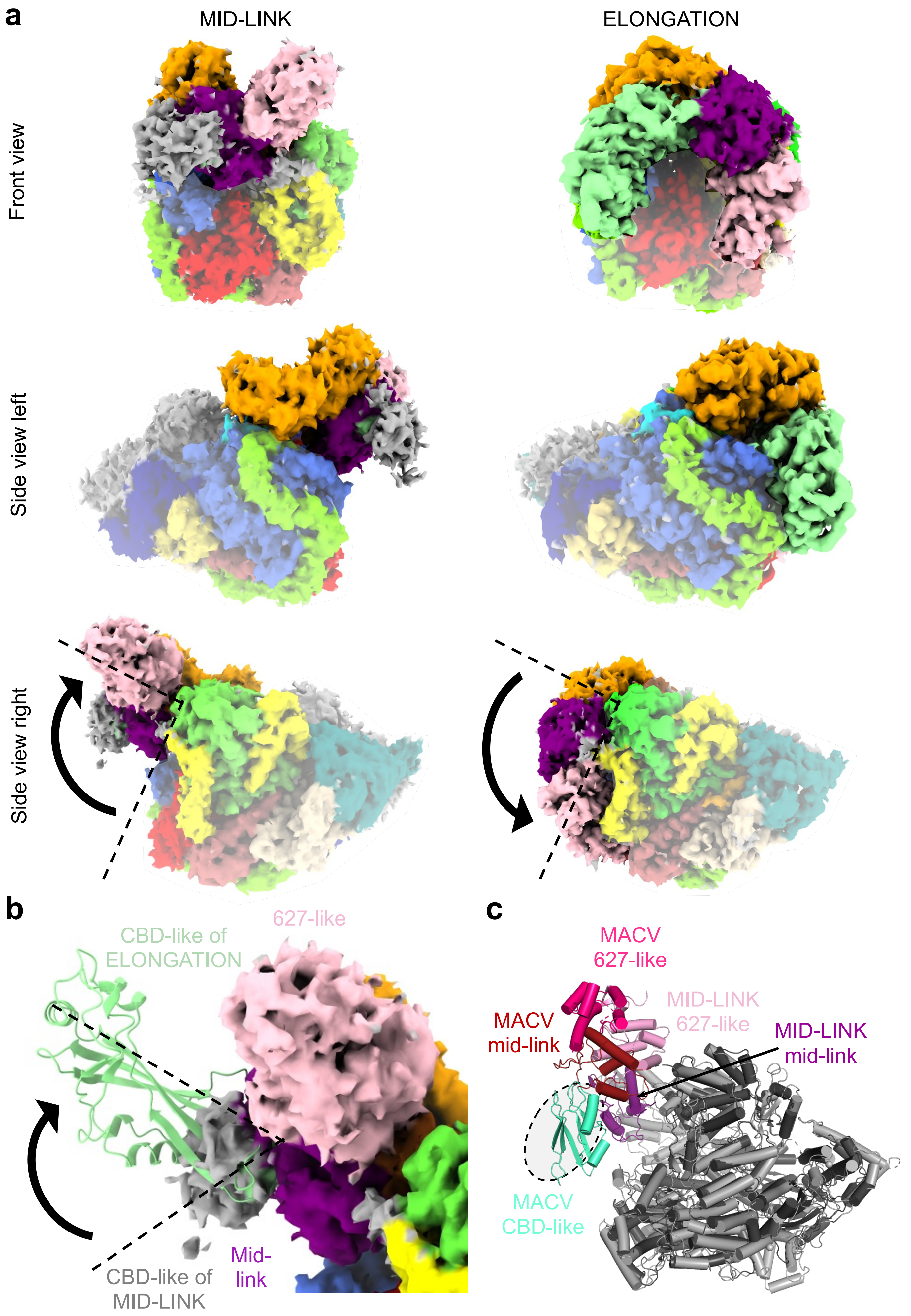

Supplementary Figure 7. Conformation of the C-terminal domains. a, Colored cryo-EM maps of MID-LINK and ELONGATION structures from three perspectives highlighting the position of the C-terminal mid-link (violet), CBD-like (palegreen) and 627-like (pink) domains. Other domains are colored as in Fig. 3. Possible rotation of the C-terminal domains in respect to the core is indicated. **b,** Rotation of the CBD-like domain (palegreen) relative to the mid-link (violet) domain is indicated. Mid-link and 627-like domains of MID-LINK and ELONGATION have been superposed and CBD-like domain of the ELONGATION structure is shown as ribbon diagram. Density in the cryo-EM map for the CBD-like domain in the MID-LINK structure is visible in grey. **c,** Similarity of the conformation of the CBD-like domain (palegreen) in MACV L protein (light grey, PDB:6KLH) and the LASV MID-LINK structure (dark grey), where the position of the CBD-like domain at low resolution is marked by a dashed circle. Mid-link, CBD-like and 627-like domains are shown in color (violet and pink for LASV, dark red, cyan and hotpink for MACV).

Supplementary Fig. 8

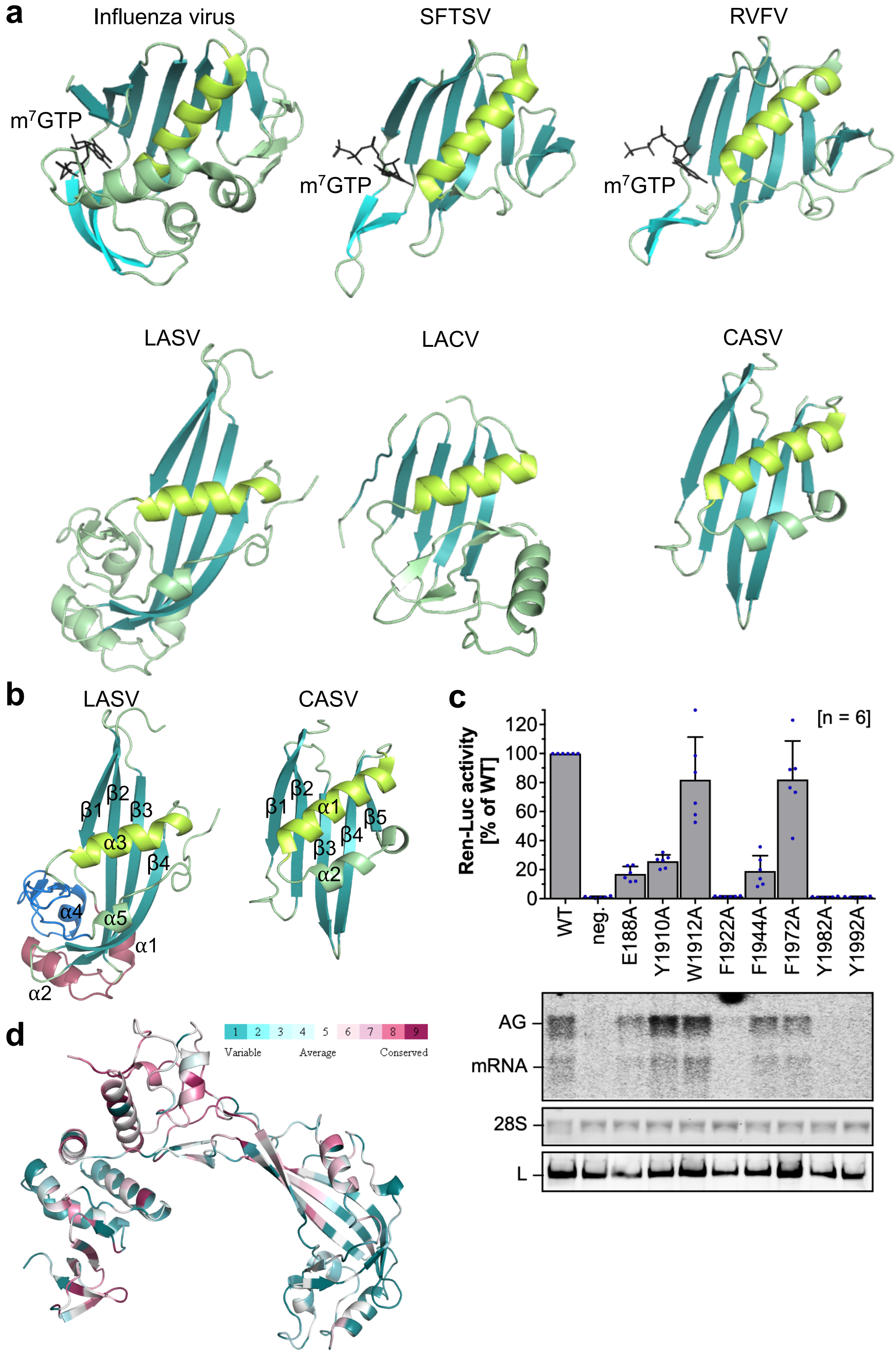

Supplementary Figure 8. (Putative) cap-binding domains. a, Comparison between different putative and functional CBDs: Influenza virus (PDB:2VQZ), SFTSV (PDB:6XYA), RVFV (PDB:6QHG), LACV (PDB:6Z8K), CASV (PDB:5MUS) and LASV (ELONGATION). Corresponding secondary structure elements of ribbon diagrams are displayed in the same colors. **b,** Side-by-side comparison between reptarenavirus (CASV) and mammarenavirus (LASV, ELONGATION structure) CBD-like domains. Insertions of the LASV domain compared to the CASV domain are colored in blue and red. Secondary structure elements are labelled. **c,** LASV mini-replicon data for L proteins with mutations of aromatic residues potentially involved in cap-binding presenting luciferase reporter activity (in standardized relative light units relative to the wild-type L protein (WT)). Data are presented as mean values +/- SD of 6 biological replicates (n=6). All biological replicates are shown as blue dots (top panel). Middle panels present Northern blotting results with signals for antigenomic viral RNA (AG), viral mRNA (mRNA) and 28S ribosomal RNA (28S) as a loading control, and bottom panel shows Western blot detection of FLAG-tagged L proteins (L) to demonstrate general expressibility of the mutants. Source data are provided as a Source Data file. **d,** Conservation of the C-terminal domain within arenaviruses according to the Supplementary Alignment file illustrated using the ConSurf web server [1]. Color legend is provided.

Supplementary Fig. 9

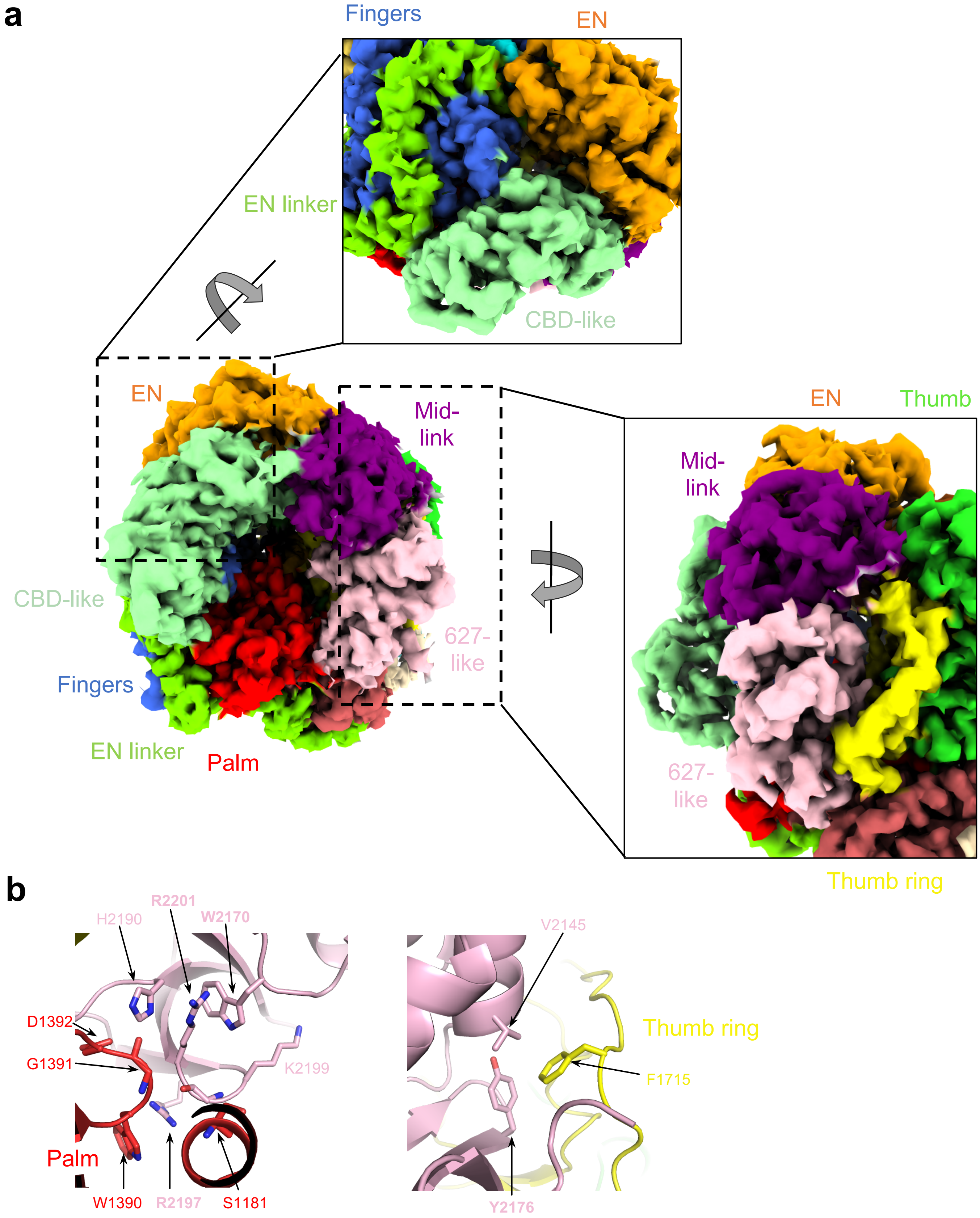

Supplementary Figure 9. Domain interactions of the C-terminal region. a, The cryo-EM map of the ELONGATION structure is presented with colors of domains according to Fig. 3. In close-ups of different perspectives, the neighboring domains of the mid-link, 627-like and CBD-like domains are visible. **b,** Close-ups of the interaction between residues identified as selectively important for viral transcription [2] of the 627-like domain with the palm (red) and thumb-ring (yellow) domains. Important main and side chains are shown as sticks with respective labels.

Supplementary Fig. 10

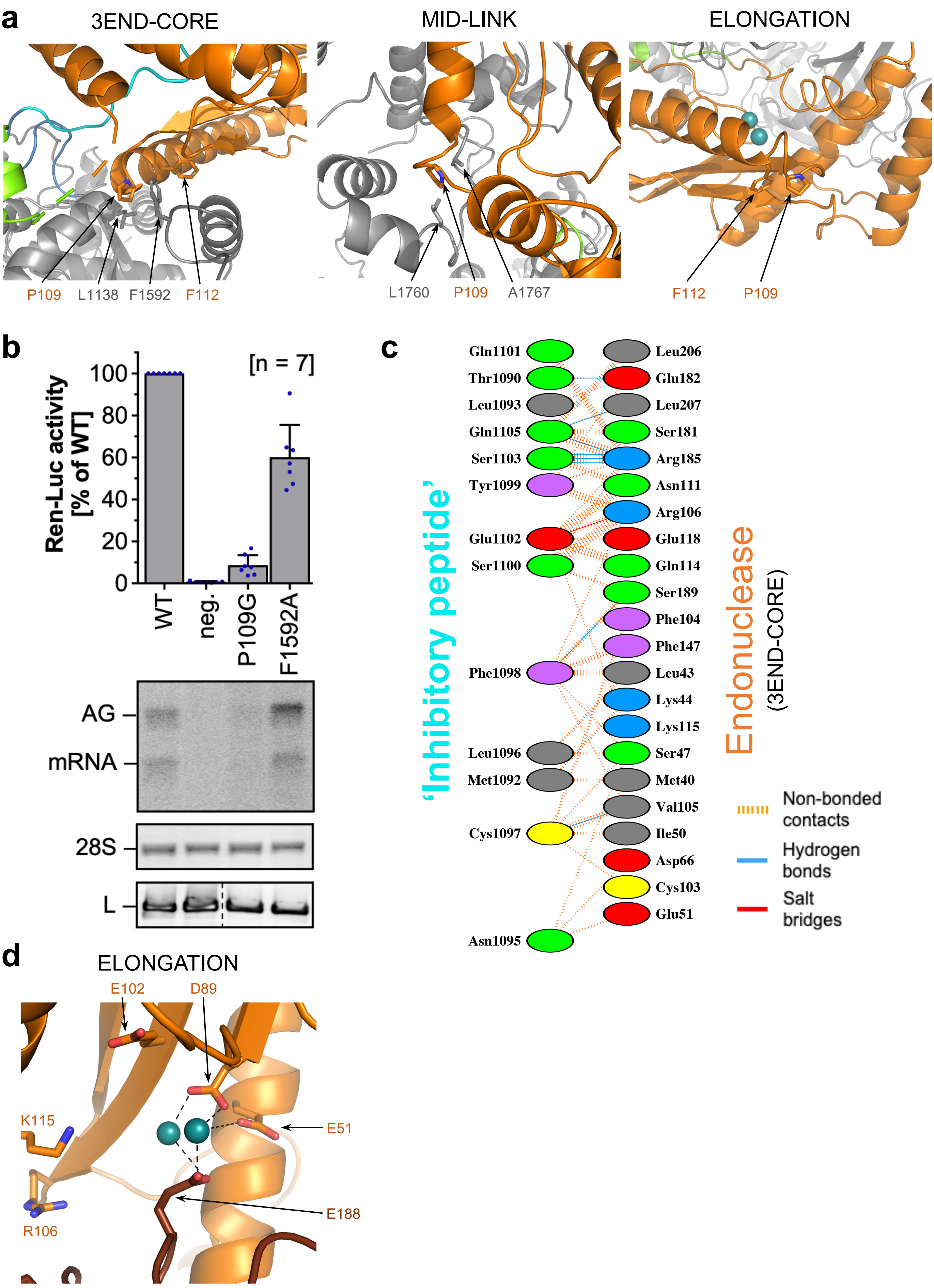

Supplementary Figure 10. Interactions of the EN domain. a, Close-ups of the interactions of residue P109 in the 3END-CORE, MID-LINK and ELONGATION structures with important side chains shown as sticks, labelled respectively. **b,** LASV mini-replicon data for L proteins with mutations P109A and F1592A presenting luciferase reporter activity (in standardized relative light units relative to the wild-type L protein (WT)). Data are presented as mean values +/- SD of 7 biological replicates (n=7). All biological replicates are shown as blue dots (top panel). Middle panels present Northern blotting results with signals for antigenomic viral RNA (AG), viral mRNA (mRNA) and 28S ribosomal RNA (28S) as a loading control, and bottom panel shows Western blot detection of FLAG-tagged L proteins (L) to demonstrate general expressibility of the mutants. Source data are provided as a Source Data file. **c,** Ligplot diagram [3] of the interface between the inhibitory peptide and the EN domain in the 3END-CORE structure generated by PDBsum [4]. **d,** Close-up of the EN active site in the ELONGATION structure showing residues E188, E51 and D89 co-ordinating the divalent metal ions. Structure is shown as a ribbon diagram, important residues as sticks and with respective labels.

Supplementary Fig. 11

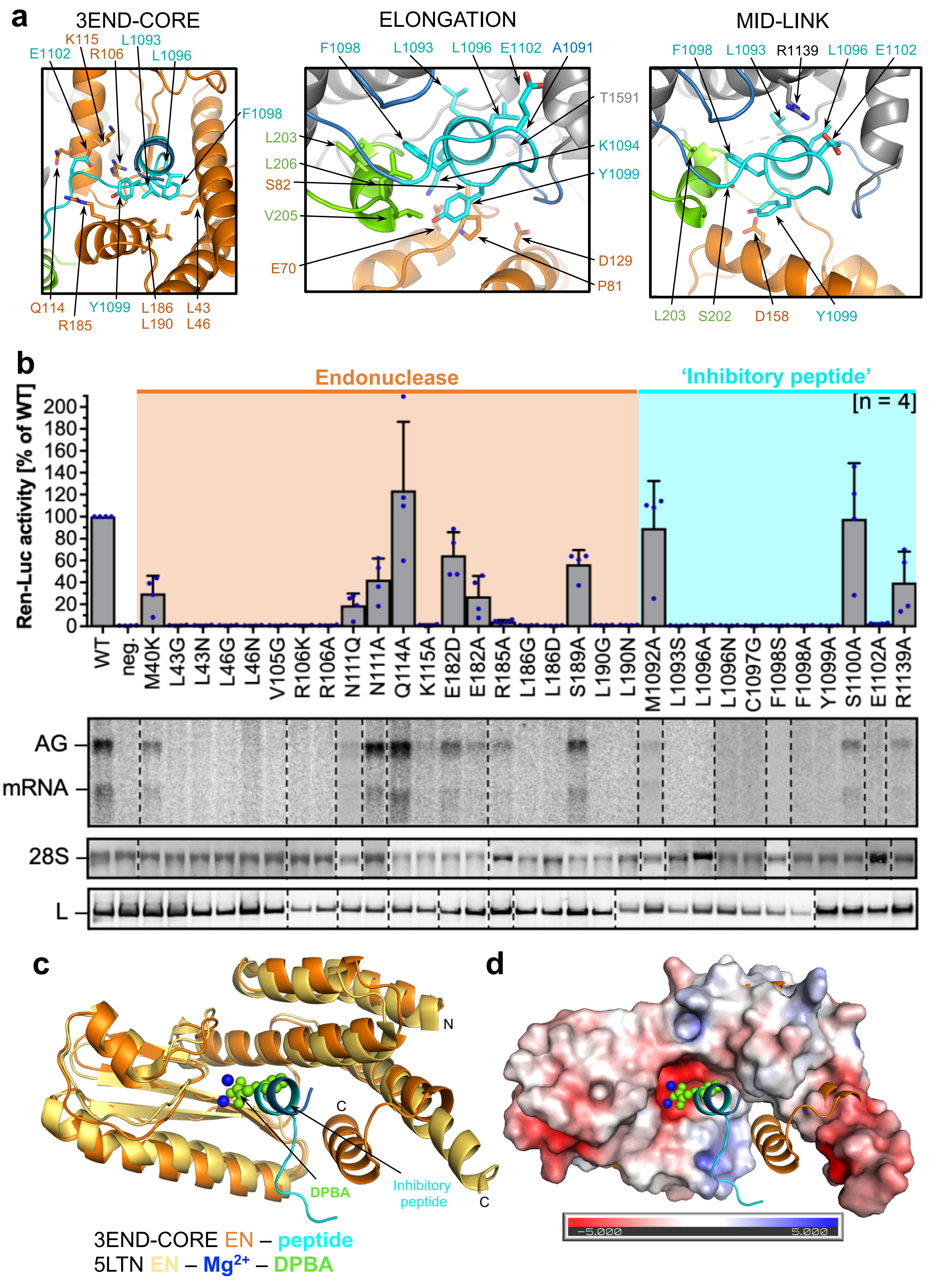

Supplementary Figure 11. The EN and the inhibitory peptide. a, Close-ups of the inhibitory peptide (cyan) and interacting residues in the 3END-CORE, ELONGATION and MID-LINK structures. EN is shown in orange, EN linker in green. **b,** LASV mini-replicon data for L proteins with mutations of residues involved in the interface between EN (orange background) and the inhibitory peptide (cyan background) (comp. Supplementary Fig. 10) presenting luciferase reporter activity (in standardized relative light units relative to the wild-type L protein (WT)). Data are presented as mean values +/- SD of 4 biological replicates (n=4). All biological replicates are shown as blue dots (top panel). Middle panels present Northern blotting results with signals for antigenomic viral RNA (AG), viral mRNA (mRNA) and 28S ribosomal RNA (28S) as a loading control, and bottom panel shows Western blot detection of FLAG-tagged L proteins (L) to demonstrate general expressibility of the mutants. Source data are provided as a Source Data file. **c,** Superimposition of the 3END-CORE EN domain (orange) with the crystal structure of Lymphocytic choriomeningitis virus (LCMV) EN domain (PDB:5LTN, yellow) solved in complex with the specific inhibitor 2,4-dioxo-4-phenylbutanoic acid (DPBA, green) bound to the EN. DPBA and the inhibitory peptide (cyan) are occupying the same space, i.e. the EN active site. Divalent metal ions (blue) and DPBA are shown as spheres. **d,** Superimposition as in (c) but with the LCMV structure shown as surface electrostatics calculated by APBS electrostatics plugin within PyMOL (Schrödinger).

Supplementary Fig. 12

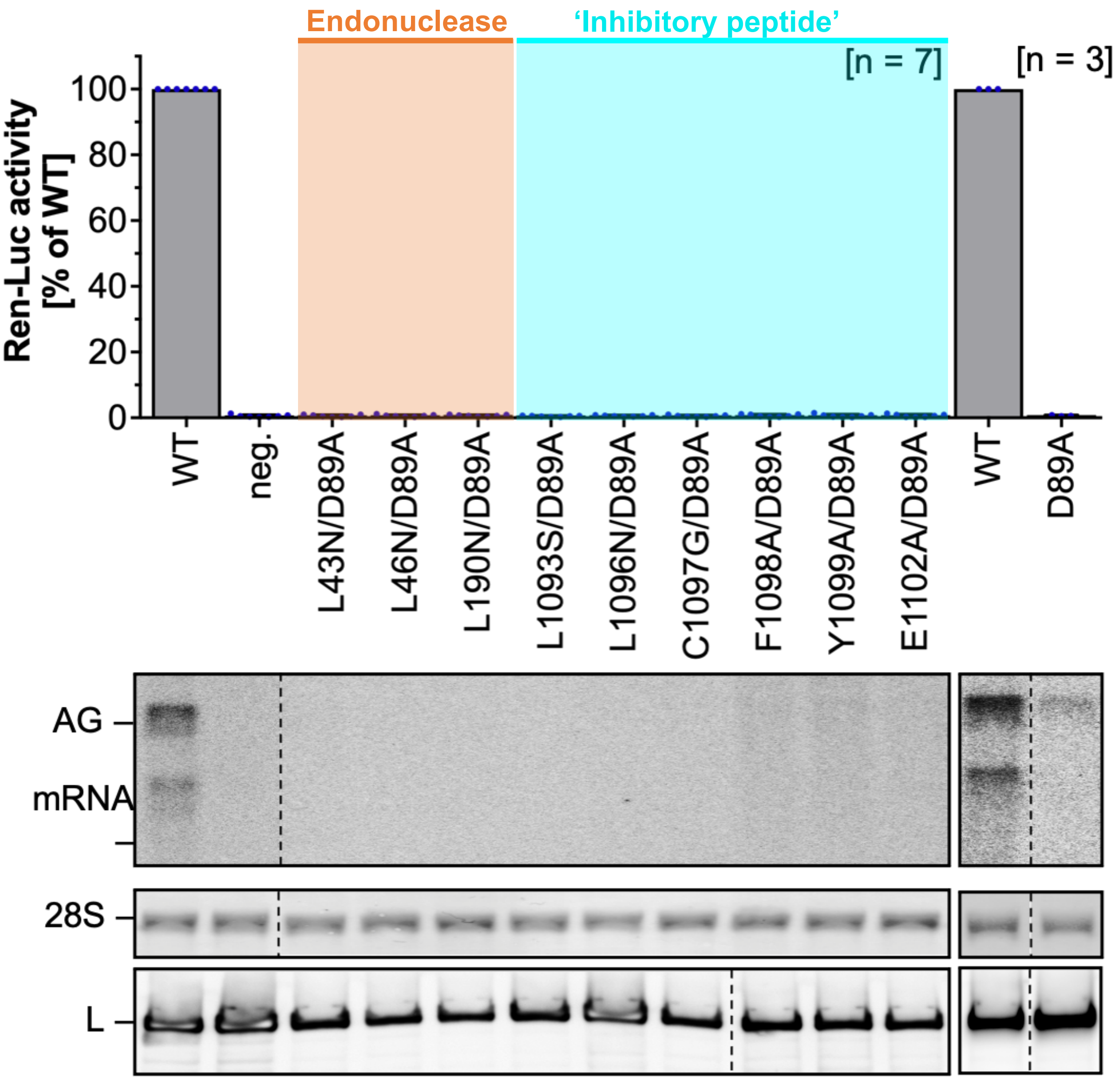

Supplementary Figure 12. Mutations of the interface of EN and the inhibitory peptide in combination with EN active site mutation D89A. LASV mini-replicon data for L proteins with double mutations of residues involved in the interface between EN (orange background) and the inhibitory peptide (cyan background) as well as EN active site mutation D89A presenting luciferase reporter activity (in standardized relative light units relative to the wild-type L protein (WT)). Data are presented as mean values +/- SD of 7 biological replicates (n=7), except for the right side of the graph, mutant D89A and the respective wildtype L, where n=3. All biological replicates are shown as blue dots (top panel). Middle panels present Northern blotting results with signals for antigenomic viral RNA (AG), viral mRNA (mRNA) and 28S ribosomal RNA (28S) as a loading control, and bottom panel shows Western blot detection of FLAG-tagged L proteins (L) to demonstrate general expressibility of the mutants. Source data are provided as a Source Data file.

Supplementary Fig. 13

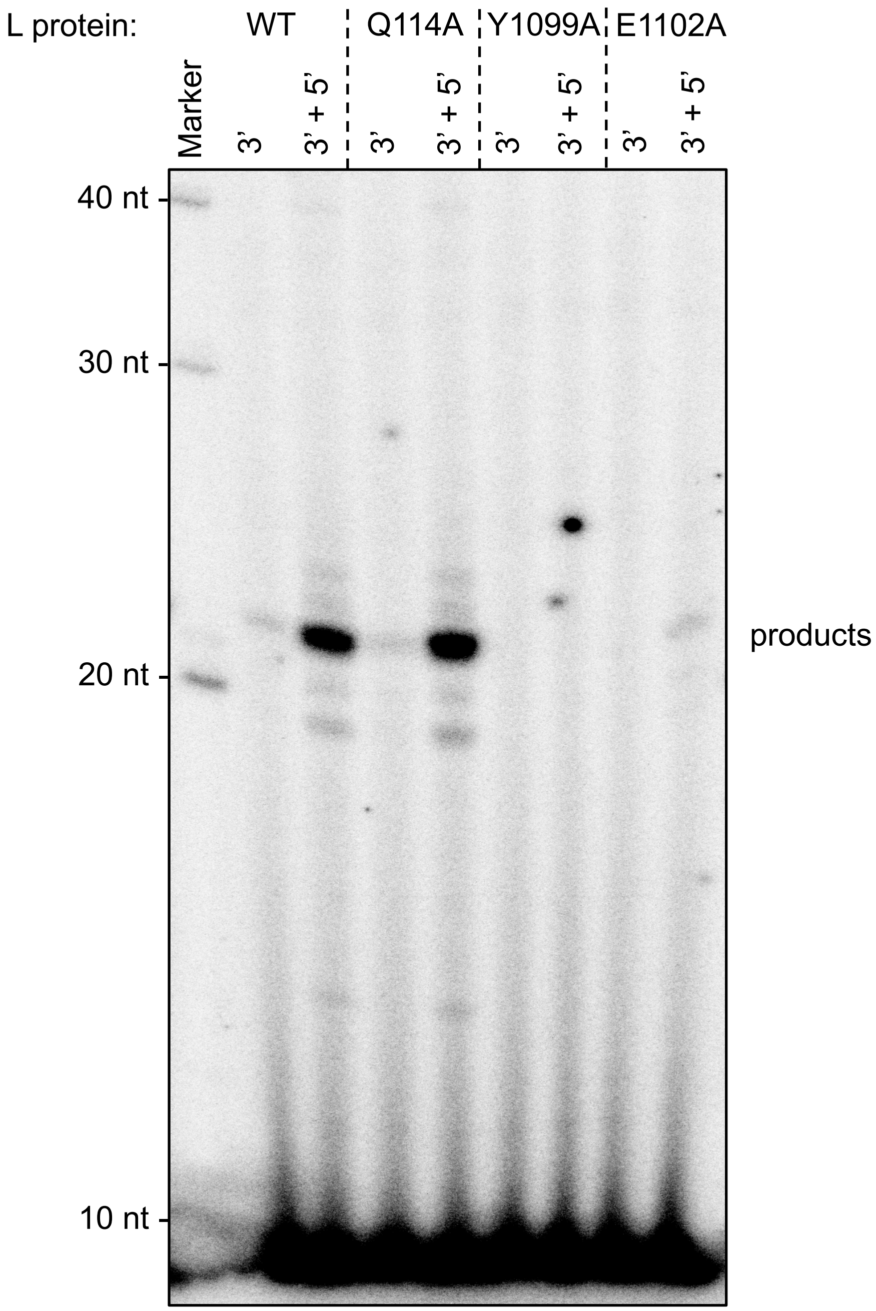

Supplementary Figure 13. ***In vitro*** **polymerase activity of selected L protein mutants.** The influence of the mutations Q114A in the EN, and Y1099A and E1102A in the inhibitory peptide on the polymerase activity of purified LASV L was tested *in vitro* and compared to the wild-type L protein (WT). The reactions were carried out with only the 3' vRNA (nts 1-19) present or with the 3' vRNA (nts 1-19) Pi and 5' vRNA (nts 0-19) Pi under standard polymerase assay conditions (see Methods). Products were separated by denaturing gel electrophoresis and visualized by autoradiography.

Supplementary Fig. 14

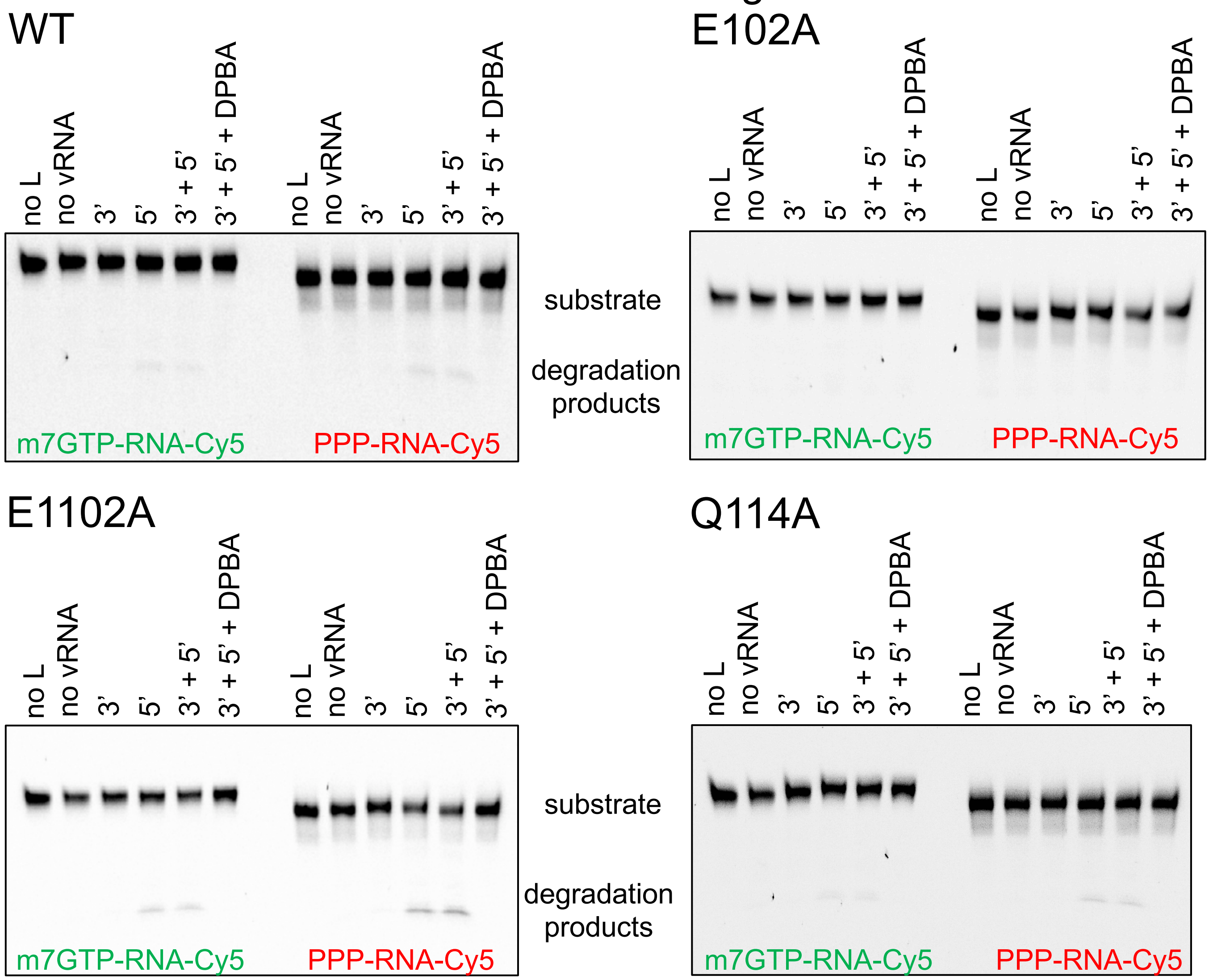

Supplementary Figure 14. ***In vitro*** **endonuclease activity of selected L protein mutants.** The ability to degrade 3' Cy5 labelled RNA with a 5' Cap (m7GTP-RNA-Cy5) or a 5' triphosphate (PPP-RNA-Cy5) was tested for wild-type LASV L (WT), the mutant E1102A in the inhibitory peptide and the EN mutant Q114A. 2,4-Dioxo-4-Phenylbutanoic Acid (DPBA), known to inhibit viral endonucleases and the endonuclease inactive mutant E102A served as negative controls. 500 nM of the respective LASV L was incubated with ~0.3 µM of the fluorescently labelled RNA substrates (Supplementary Table 1) in assay buffer at 37°C for 2h (see Methods). Reaction products were separated on a denaturing polyacrylamide gel and fluorescence signals were detected with a VILBER LOURMAT FUSION SL4 imaging system using the Starlight Module with an excitation wavelength of 624 nm and a 695 nm emission filter.

Supplementary Fig. 15

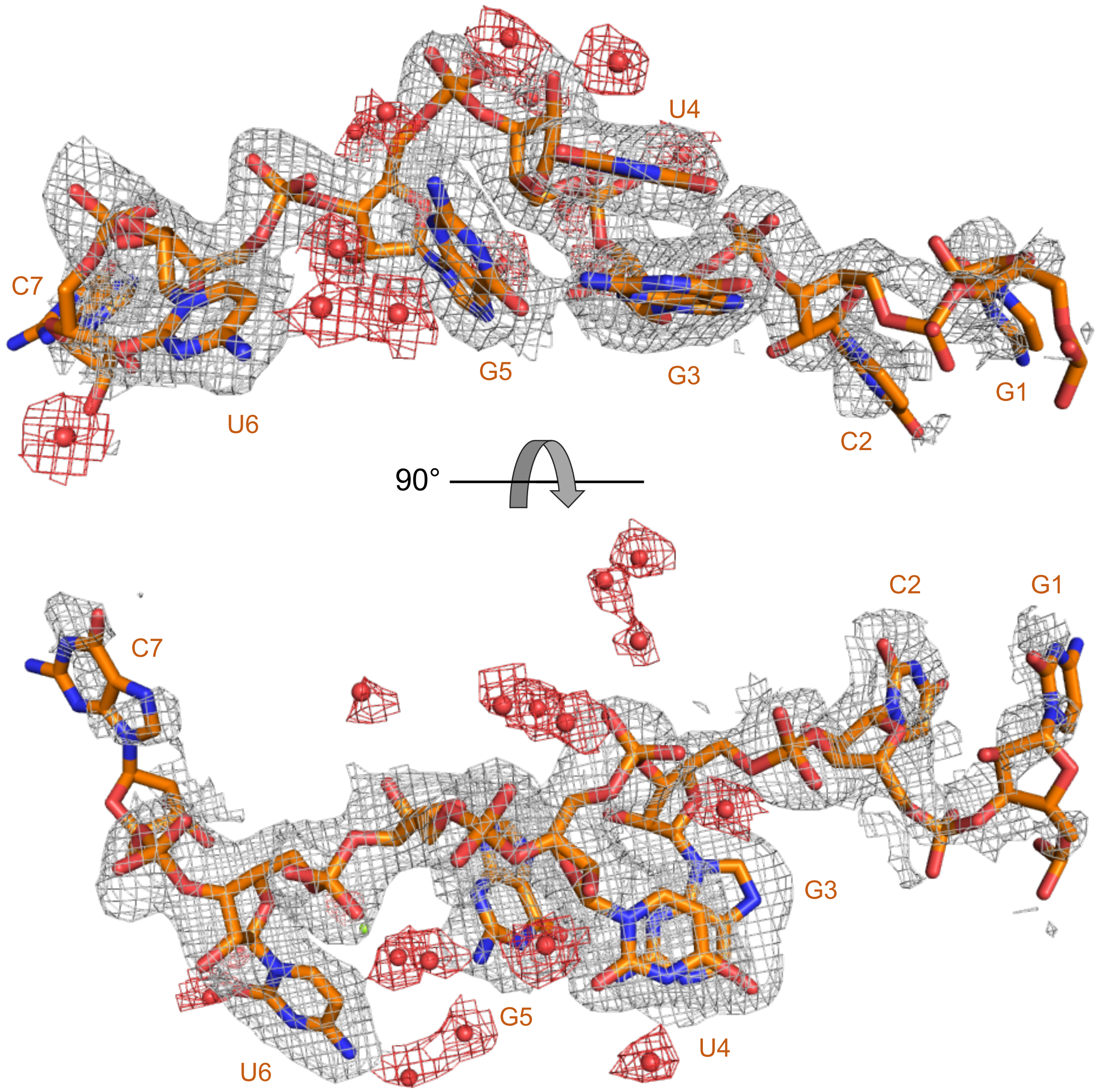

Supplementary Figure 15. 3' RNA bound to the secondary binding site. Experimental map (grey mesh) of the 3' RNA (orange sticks) and the water molecules (red mesh and spheres) in the 3END-CORE structure from two perspectives as indicated.

Supplementary Fig. 16

a

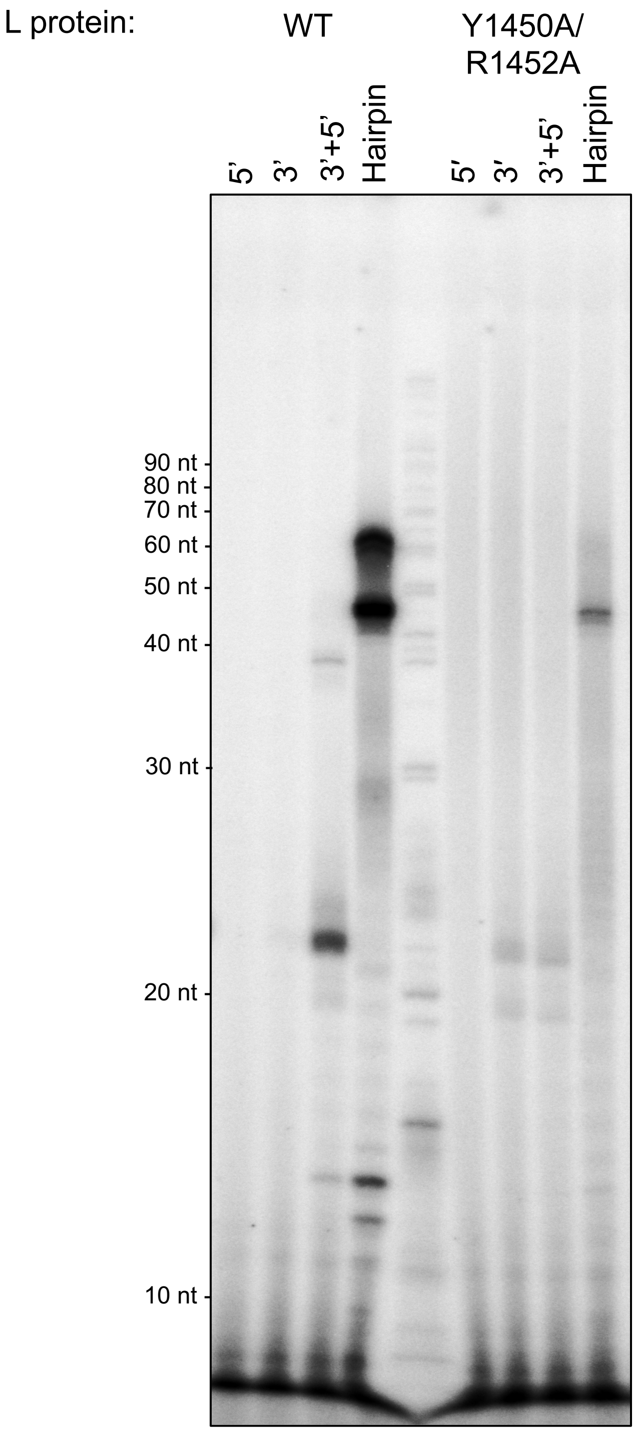

b

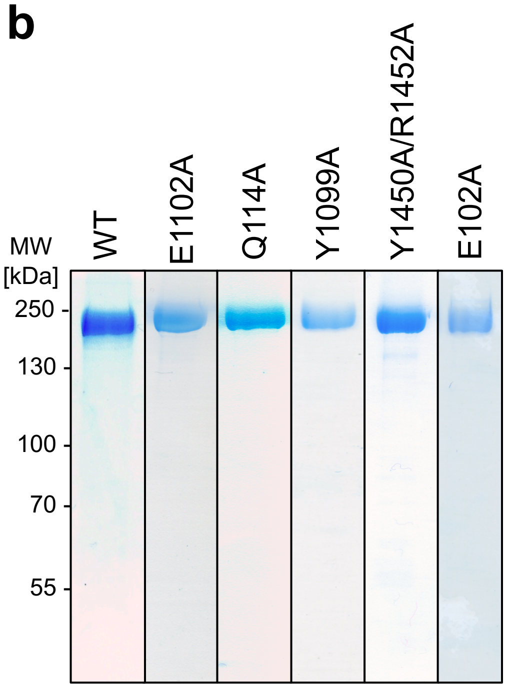

Supplementary Figure 16. ***In vitro*** **activity of the wild-type L protein vs. mutant Y1450A/R1452A. a,** The influence of the double-mutation Y1450A/R1452A of the 3' vRNA secondary binding site on the polymerase activity of purified LASV L was tested *in vitro* and compared to the wild type (WT). The reactions were carried out under standard polymerase assay conditions (see Methods) either with (i) only the 3' vRNA (nts 1-19) Pi or (ii) 5' vRNA (nts 0-19) Pi present, (iii) together with both 3' and 5' vRNAs or (iv) in the presence of a 47 nt hairpin RNA containing the connected 3' and 5' promoter sequences. Products were separated by denaturing gel electrophoresis and visualized by autoradiography. **b,** SDS-PAGE analysis of the purified LASV L proteins.

Supplementary Fig. 17

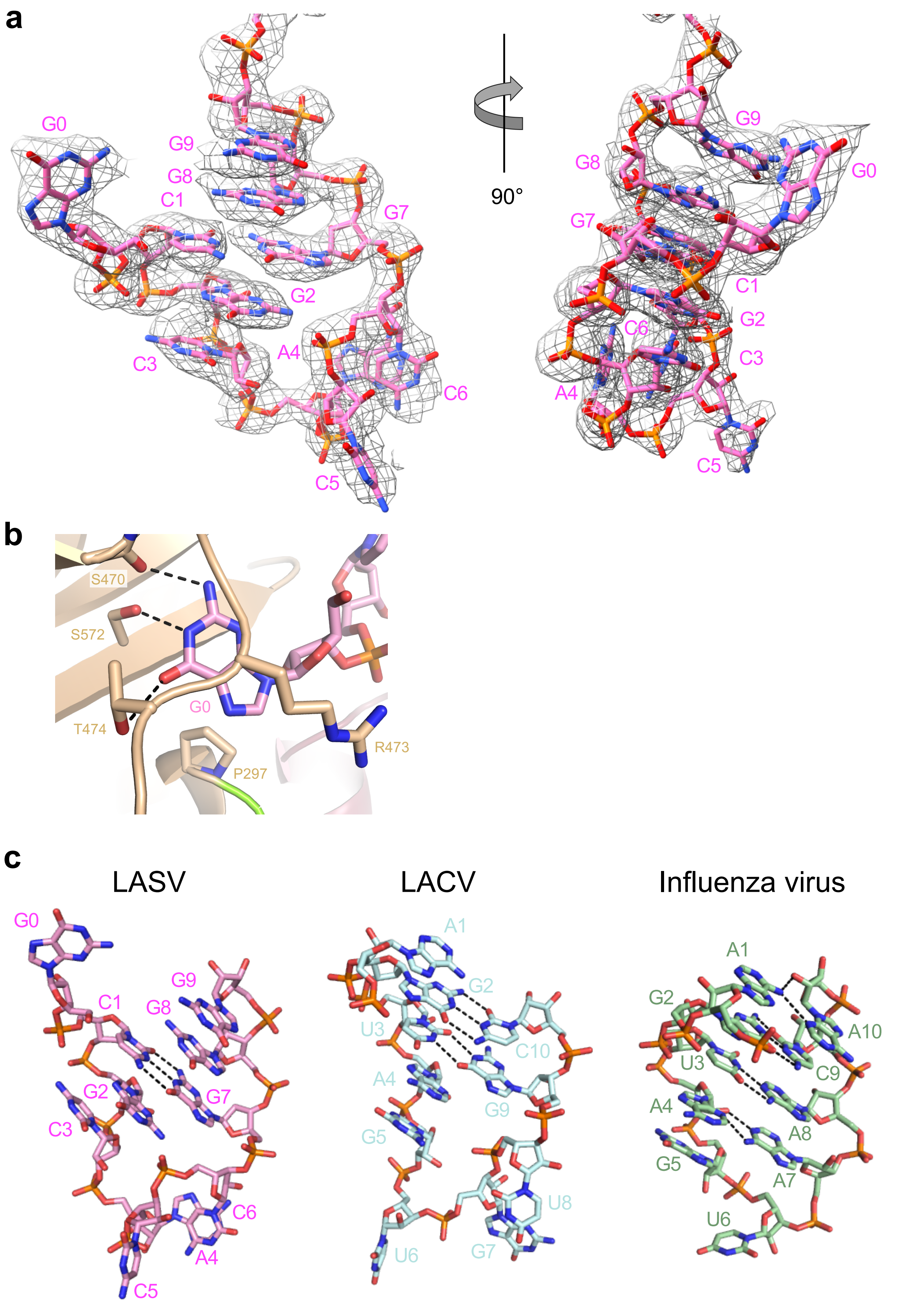

Supplementary Figure 17. 5' vRNA hook. a, Experimental map of the 5' vRNA hook (grey mesh) around the RNA shown as sticks from two perspectives as indicated. **b,** Close-up of the interaction between the pyramid base and the G0 nucleotide of the 5' vRNA. Hydrogen bonds are indicated by dotted lines. Important main and side chains are shown as sticks with respective labels. **c,** Comparison between the 5' vRNA hook structures of LASV (nts 0-9, PRE-INITIATION, pink), LACV (nts 1-10, PDB: 6Z6G, blue), and influenza virus (nts 1-10, PDB: 6T0V, green) presented as sticks. Hydrogen bonds between bases are shown as dotted black lines and bases are labelled.

Supplementary Fig. 18

a

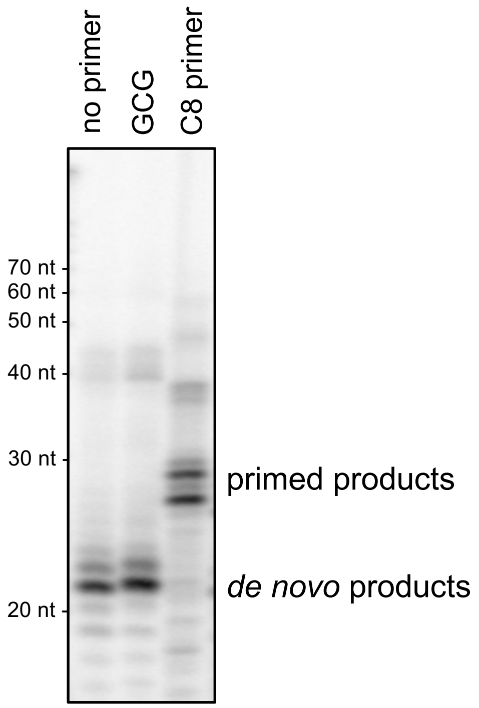

b

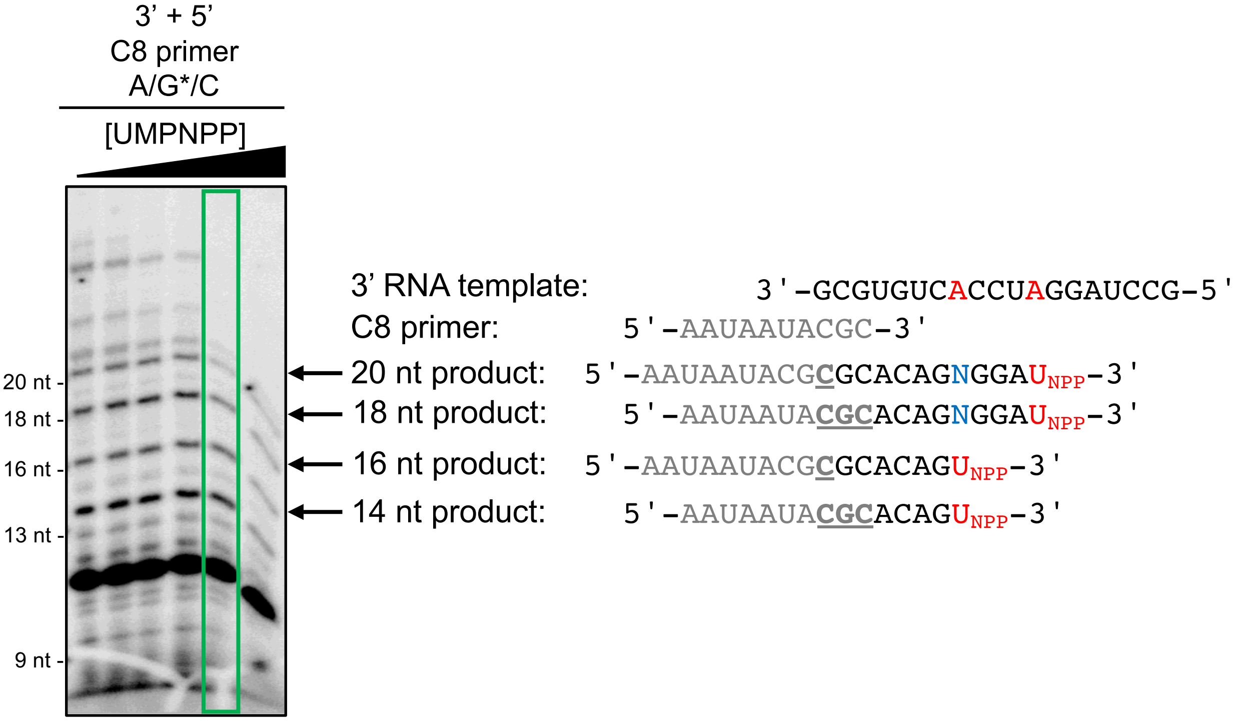

Supplementary Figure 18. Primed RNA synthesis by LASV L protein. a, The influence of the tri-nucleotide GCG and the C8 primer (Supplementary Table 1) on the polymerase activity of purified LASV L was tested *in vitro* compared to the unprimed (*de novo*) reaction. The reactions were carried out in presence of both 3' vRNA (nts 1-19) Pi and 5' vRNA (nts 0-19) Pi under standard polymerase assay conditions (see Methods). Products were separated by denaturing gel electrophoresis and visualized by autoradiography. **b,** For polymerase stalling, LASV L protein was incubated *in vitro* with C8 primer (Supplementary Table 1) and increasing amounts of the non-hydrolysable UTP analogue UMPNPP under primer-dependent polymerase assay conditions (see Methods). The reaction was started by addition of NTPs (0.25 mM GTP/ ATP/ 0.125 mM CTP and 0.03-0.25 mM UMPNPP). Products were separated by denaturing gel electrophoresis and visualized by autoradiography. Possible products are shown on the right. Positions for potentially mis-incorporated nucleotides (N) or stalling by UMPNPP (U_{NPP}) are indicated.

Supplementary Fig. 19

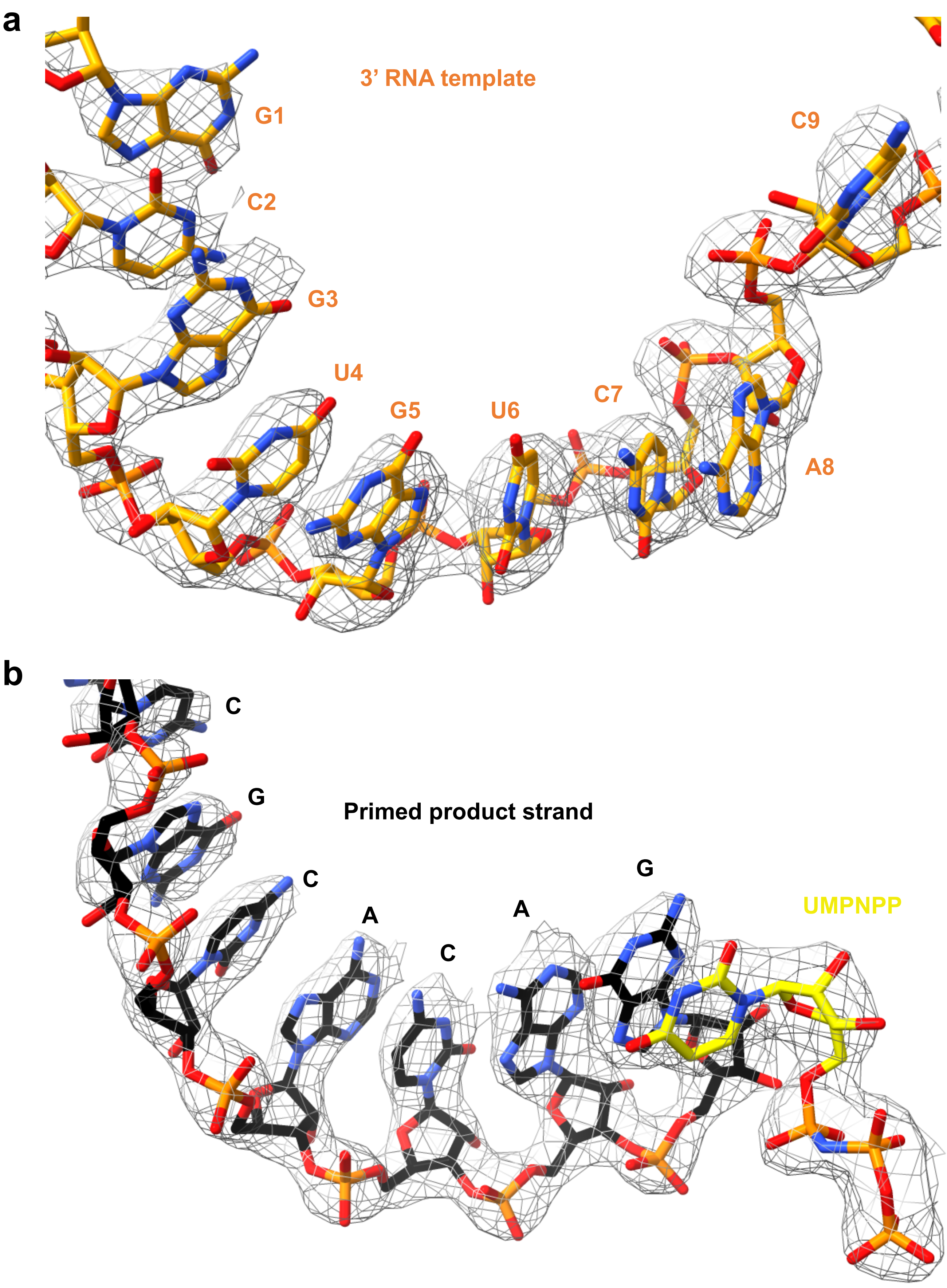

Supplementary Figure 19. Template and product RNA in the ELONGATION structure. a, Experimental map of the 3' RNA template (grey mesh) around the RNA shown as orange sticks. **b,** Experimental map of the product RNA (grey mesh) around the RNA (black) and the non-hydrolysable UTP (UMPNPP, yellow) shown as sticks.

Supplementary Fig. 20

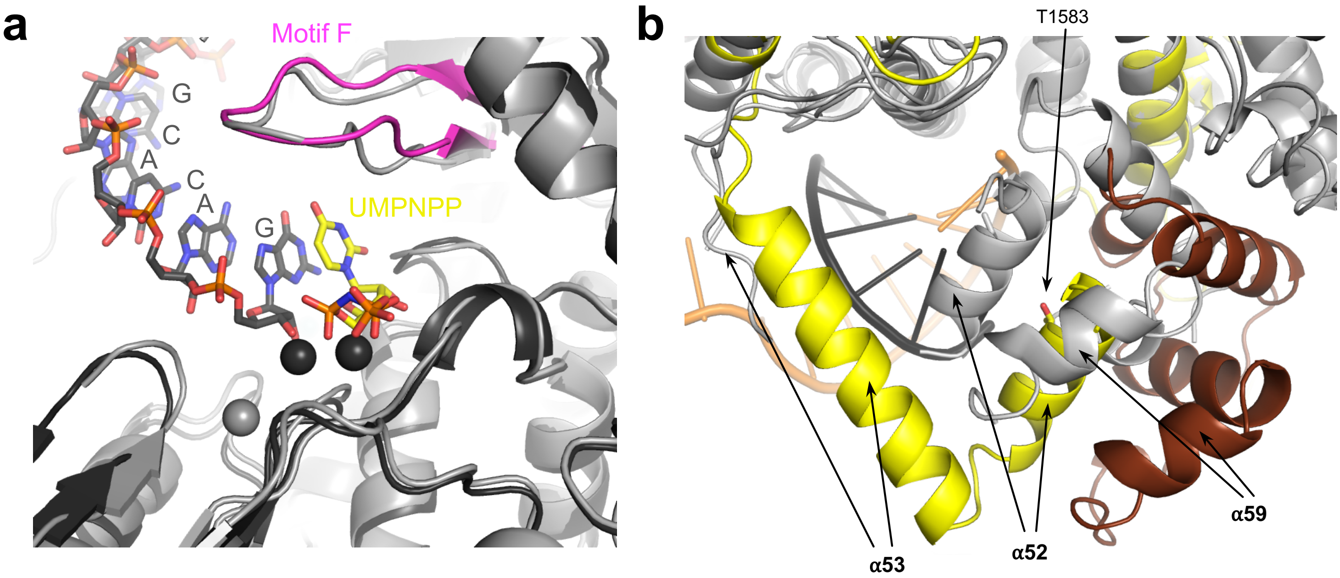

Supplementary Figure 20. Active site comparison between ELONGATION and PRE-INITIATION structures. a, Close-up of the RdRp active site of the ELONGATION (black) and PRE-INITIATION (grey) structures. Product RNA and non-hydrolysable UTP (UMPNPP) are shown as sticks. Motif F of the ELONGATION structure is highlighted in magenta. **b,** Close-up of the RdRp active site towards the product exit channel with the product RNA shown as grey ribbon. Thumb-ring and lid domains of the ELONGATION structure are shown in yellow and brown, respectively. PRE-INITIATION structure shown in grey. Corresponding α-helices 52, 53 and 59 are labelled. Residue T1583 stacking on the terminal nucleotide of the product RNA is shown as sticks.

Supplementary Fig. 21

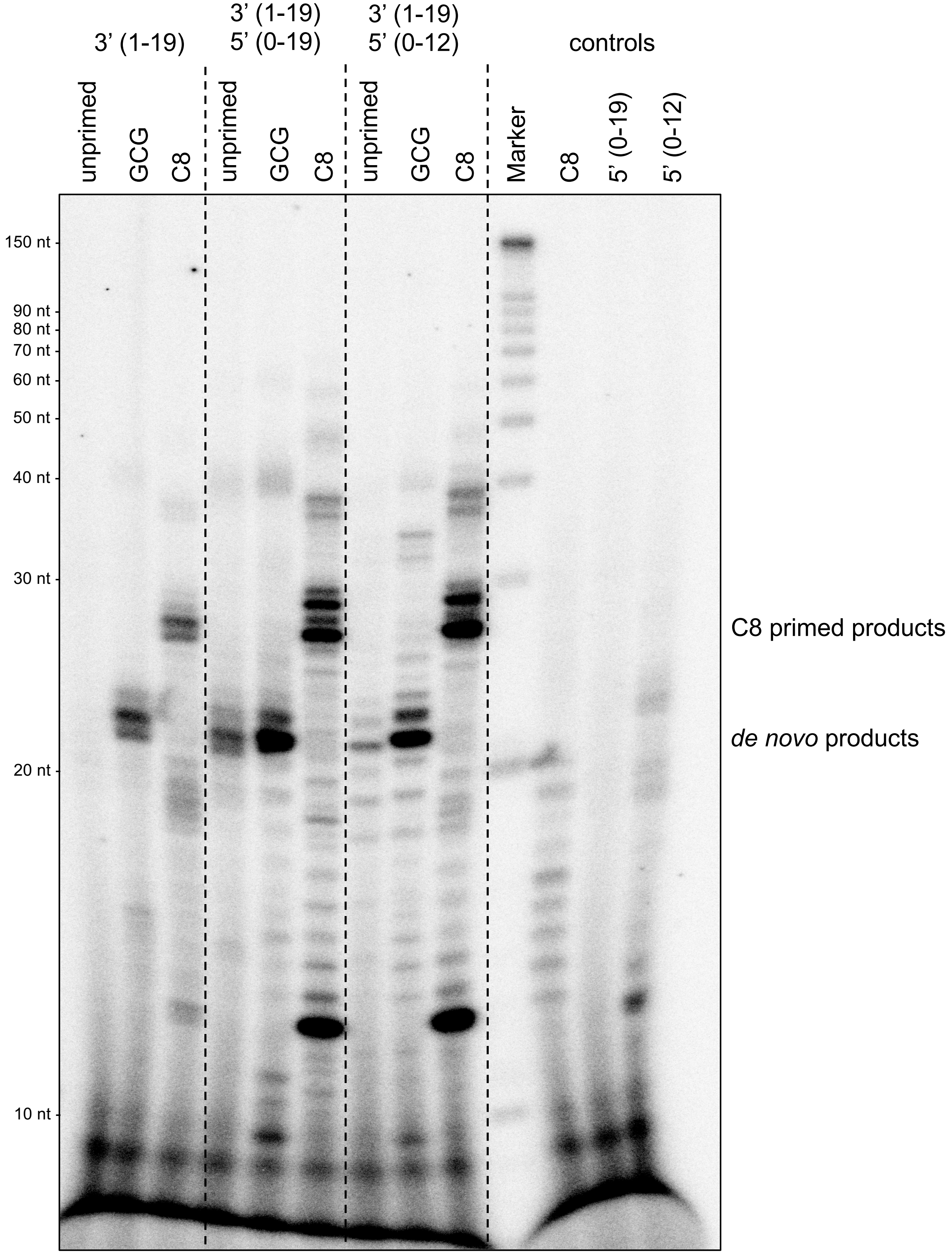

Supplementary Figure 21. Role of the 5' RNA. The influence of the 5' vRNA (nts 0-19) and 5' vRNA (nts 0-12, corresponding to the hook) on the polymerase activity of purified LASV L was tested *in vitro* for the unprimed (*de novo*) or primed reaction using the tri-nucleotide GCG or the C8 primer (Supplementary Table 1). The reactions were carried out under standard polymerase assay conditions (see Methods) either with (i) only the 3' vRNA (nts 1-19) present, (ii) together with 5' vRNA (nts 0-19) or (iii) 5' vRNA (nts 0-12). Reactions with LASV L and C8, 5' vRNA (nts 0-19) or 5' vRNA (nts 0-12) only are provided as additional controls. Products were separated by denaturing gel electrophoresis and visualized by autoradiography.

Supplementary Fig. 22

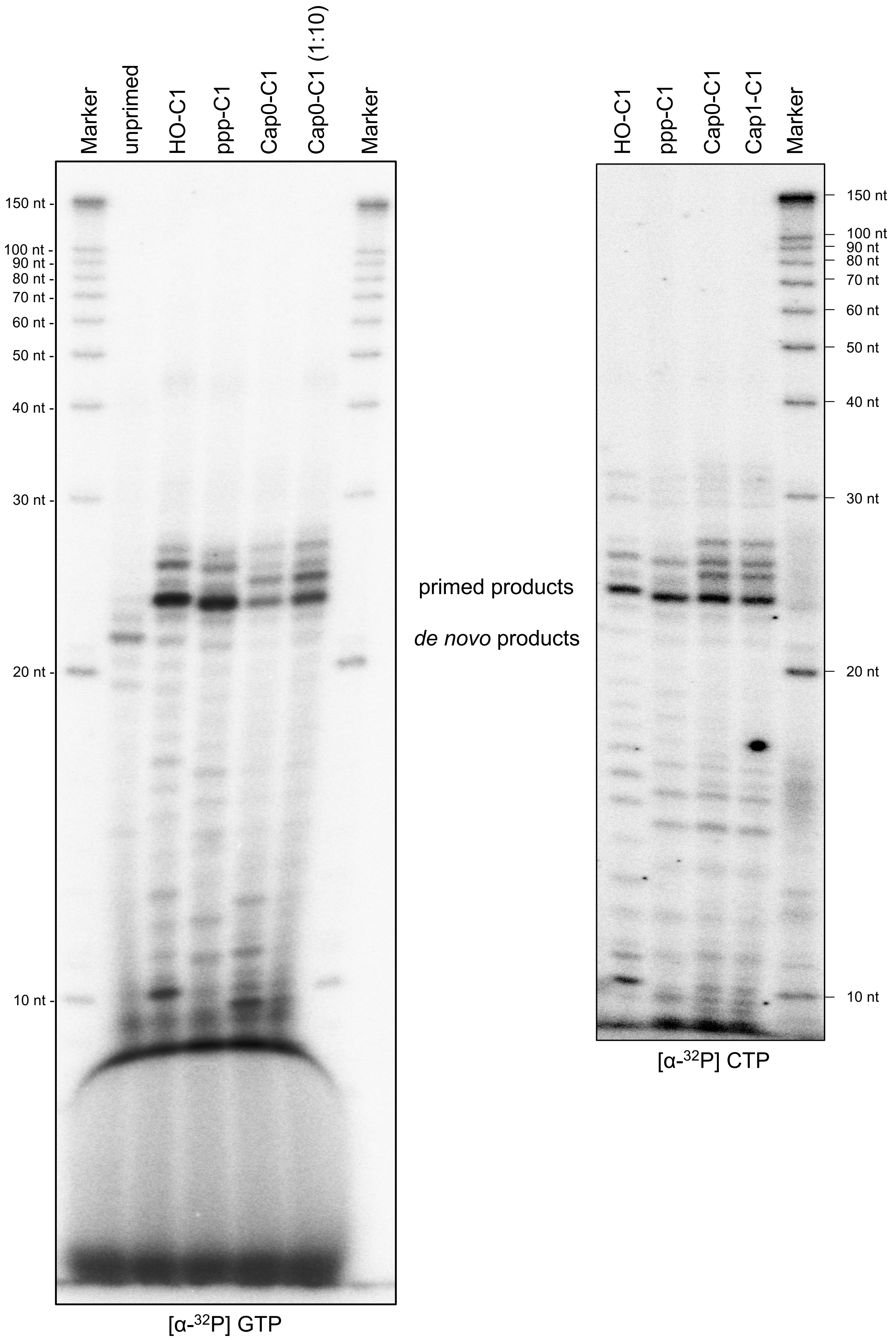

Supplementary Figure 22. ***In vitro*** **polymerase activity of LASV L in presence of capped and uncapped primers.** The influence of the 5' modification of the primer C1: 5'-OH, 5'-ppp and 5'-Cap0 or 5'-Cap1 (Supplementary Table 1) on the polymerase activity of purified LASV L was tested *in vitro* compared to the unprimed (*de novo*) reaction. The reactions were carried out in presence of both vRNAs (3' vRNA (nts 1-19) Pi and 5' vRNA (nts 0-19) Pi) under primer-dependent polymerase assay conditions (see Methods). Products were separated by denaturing gel electrophoresis and visualized by autoradiography. On the left gel, the 1:10 dilution of the Cap0-C1 primer gives more intense product bands than the undiluted primer probably as there is remaining GTP from the capping reaction (even after precipitation), which is incorporated instead of radioactive GTP. As a control we tested the same conditions with radioactive CTP instead of GTP (gel on the right side).

Supplementary Fig. 23

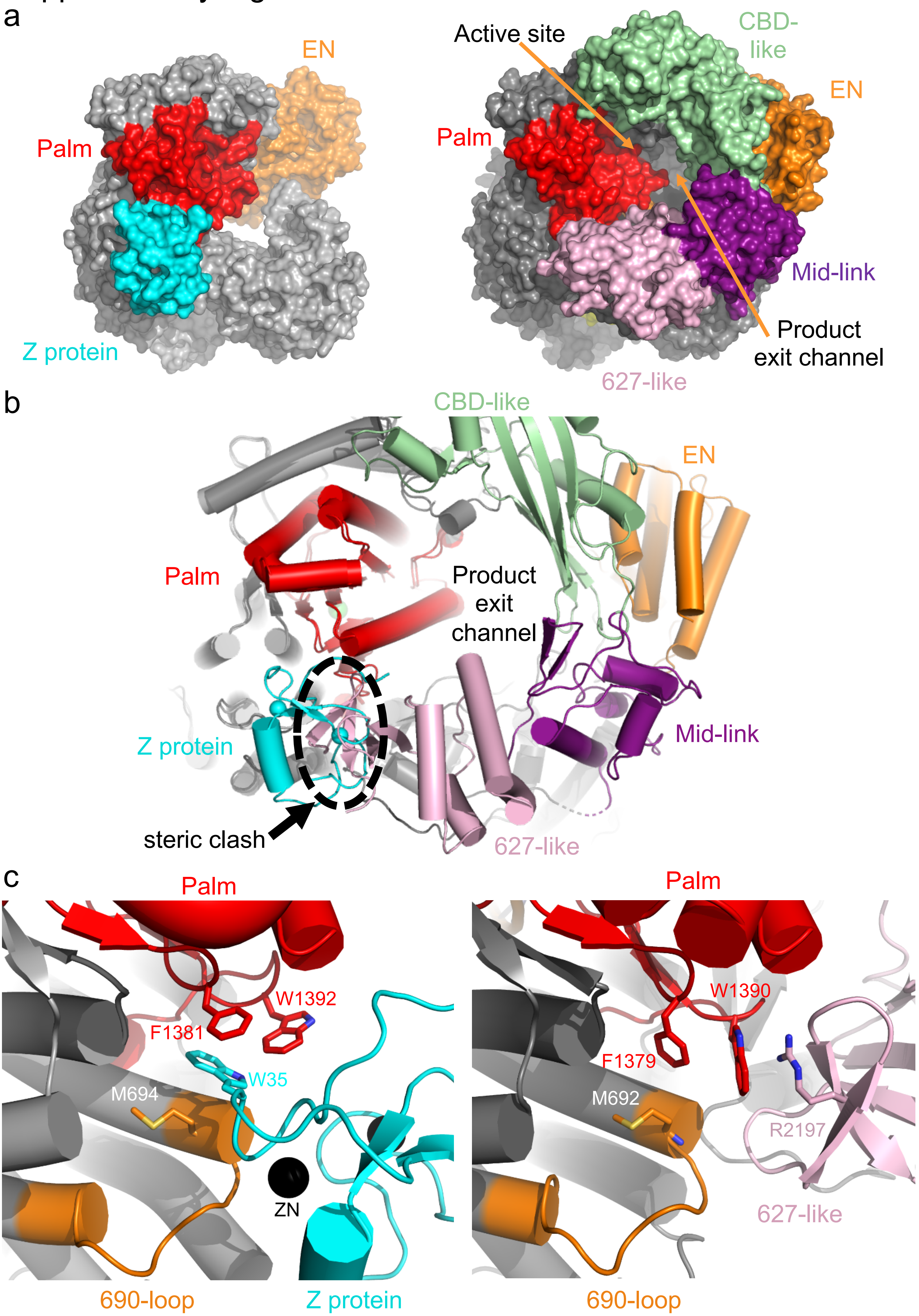

Supplementary Figure 23. Comparison of the L-Z complex with the ELONGATION structure. a, Left: Surface view of Lassa L-Z complex[5] (PDB: 7CKL) with Z protein in cyan. The L protein core is in grey with the palm domain highlighted in red and endonuclease in orange. Right: Equivalent surface view of the ELONGATION complex (this work) with the CBD-like domain in light green, the mid-link domain in violet, and the C-terminal 627-like domain in light pink. The active site and the product exit channel are indicated. The Z protein and the 627-like domain both bind to the external rim of the palm domain. **b,** Superposition of the two structures in cartoon representation highlighting the steric clash excluding simultaneous binding of the Z protein and 627-like domain to the polymerase core. **c,** Left: Interactions of Z protein W35 with residues M694, F1381 and W1392 from the 690- and 1390-loops of Lassa virus L protein (numbering for strain G3278-SLE-2013). Right: Equivalent residues for the Lassa virus L protein Bantou 289 strain. W1390 stacks against R1297 from the C-terminal 627-like domain. The conformation of the 690-loop does not change.

Supplementary Fig. 24

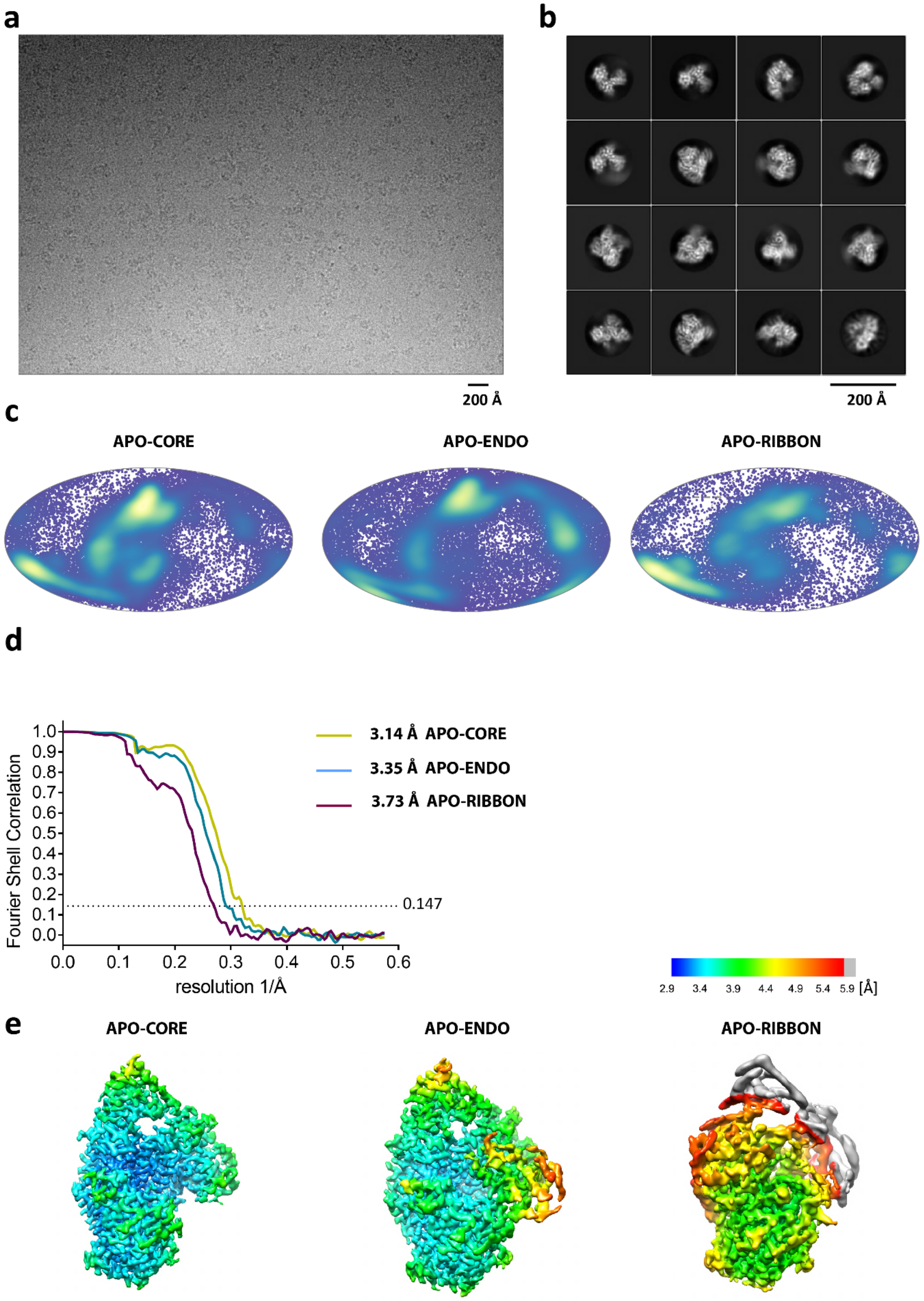

Supplementary Figure 24. Cryo-EM of APO LASV L protein

a, Representative micrograph of APO LASV L protein in free standing ice after MotionCor2[6] correction at defocus of ~2.5 µm. **b,** 2D-class averages of the APO LASV L protein complex. **c**, Angular distribution for particle projections of the APO-CORE, APO-ENDO and APO-RIBBON, respectively, visualized on a globe-like plane. **d,** Fourier shell correlation (FSC) curves for the APO-CORE (yellow), APO-ENDO (cyan) and APO-RIBBON (purple), respectively. The plot of the FSC between two independently refined half-maps shows the overall resolution of the two maps as indicated by the gold standard FSC 0.143 cut-off criteria [7]. **e,** Surface representation of local resolution distribution of the APO-CORE, APO-ENDO and APO-RIBBON, respectively. Maps are colored according to the local resolution calculated within the RELION software package[8,9]. Resolution is as indicated in the color bar.

Supplementary Fig. 25

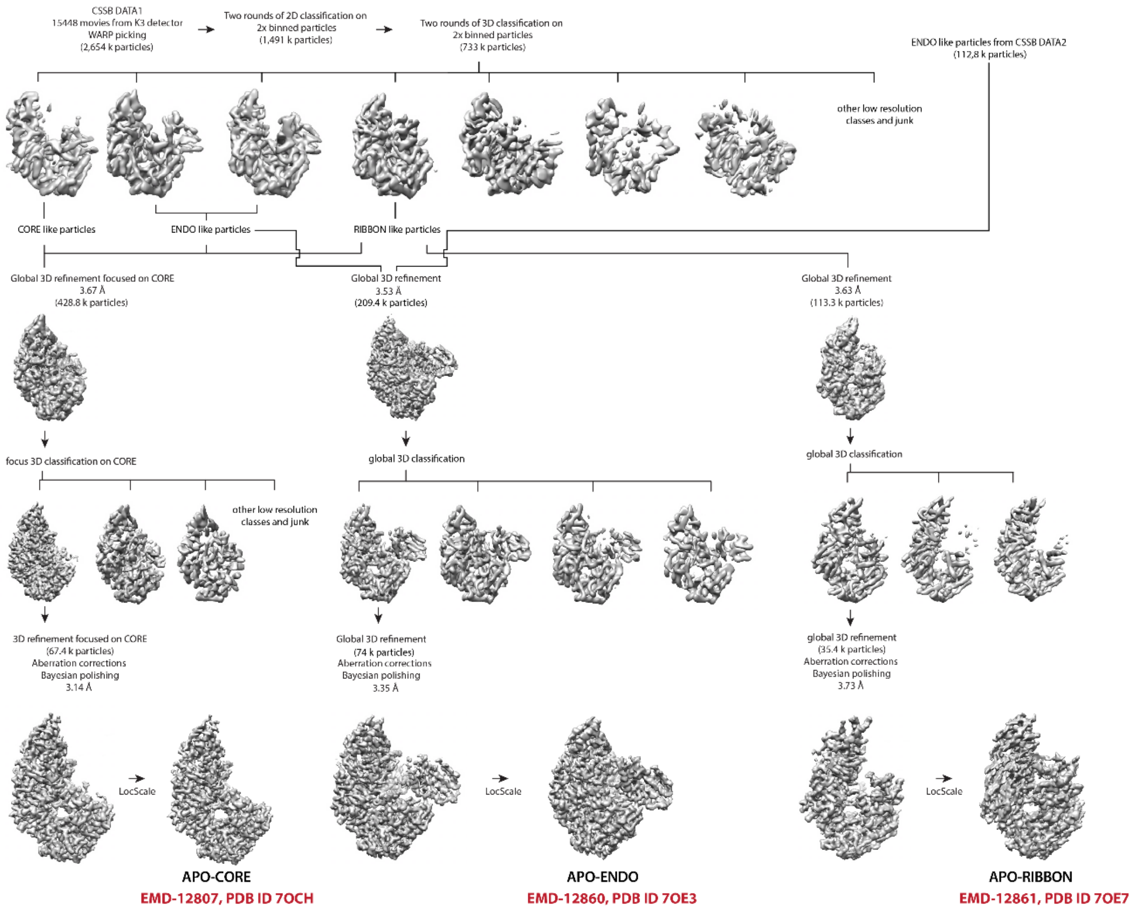

Supplementary Figure 25. Cryo-EM data 3D classification and refinement scheme of APO LASV L protein.

Summary of the cryo-EM 3D classification and refinement scheme of the APO LASV L protein. CSSB DATA1 2D classes with well-defined secondary structure features were merged (733k particles). The merged particles were classified into ten 3D classes with angular assignment. Incomplete, low resolution, and damaged particle classes were excluded from further data analyses. Three most prominent 3D classes of the APO LASV L protein corresponding to APO-CORE, APO-ENDO and APO-RIBBON were identified. APO-CORE was focus-3D classified around the CORE region of the LASV L protein and the best defined class was focus 3D-refined. APO-ENDO was merged with the same class from CSSB DATA2, globally 3D-refined, and further 3D classified. Best defined APO-ENDO class was globally 3D-refined. APO-RIBBON was processed similarly to APO-ENDO. Final cryo-EM maps were refined and post-processed with their respective masks in RELION 3.1 [8,9] and filtered by LocScale [10].

Supplementary Fig. 26

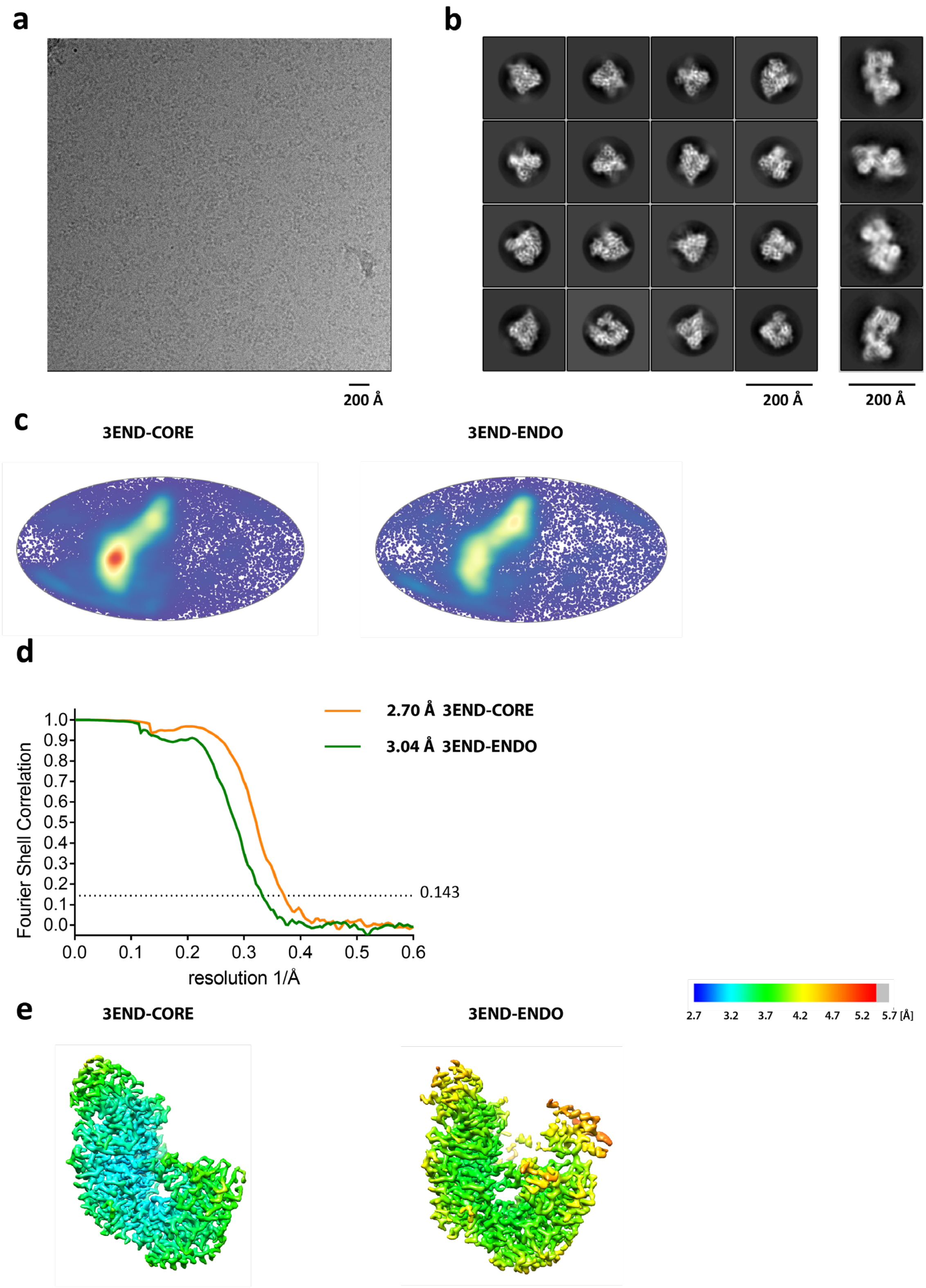

Supplementary Figure 26. Cryo-EM of LASV L protein with vRNA 3' end alone

a, Representative micrograph of LASV L protein with vRNA 3' end alone in free standing ice after MotionCor2[6] correction at defocus of ~2.5 µm. **b,** 2D-class averages of the of LASV L protein with vRNA 3' end alone - in monomeric (**left**) and dimeric (**right**) form. **c,** Angular distribution for particle projections of the 3END-CORE and 3END-ENDO, respectively, visualized on a globe-like plane. **d,** Fourier shell correlation (FSC) curves for the 3END-CORE (orange) and 3END-ENDO (green), respectively. The plot of the FSC between two independently refined half-maps shows the overall resolution of the two maps as indicated by the gold standard FSC 0.143 cut-off criteria[7]. **e,** Surface representation of local resolution distribution of the 3END-CORE and 3END-ENDO, respectively. Maps are colored according to the local resolution calculated within the RELION software package[8,9]. Resolution is as indicated in the color bar.

Supplementary Fig. 27

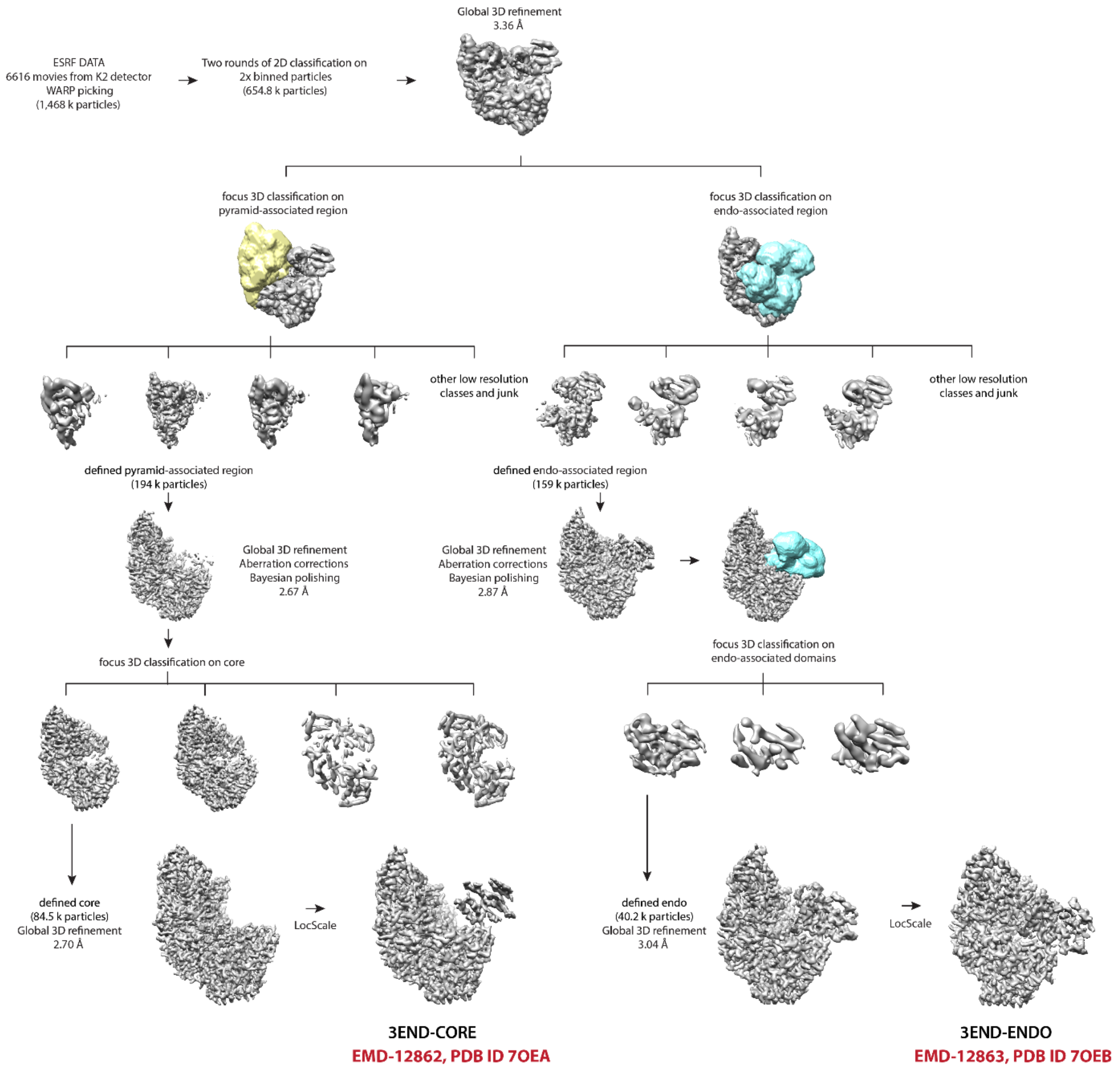

Supplementary Figure 27. Cryo-EM data 3D classification and refinement scheme of LASV L protein with vRNA 3' end alone

Summary of the cryo-EM 3D classification and refinement scheme of of LASV L protein with vRNA 3' end alone. ESRF DATA 2D classes with well-defined secondary structure features were merged (654.8k particles). The merged particles were globally 3D-refined and then in parallel focus 3D-classified around the pyramid-associated domains (**left**, yellow) and endo-associated domains (**right**, blue). The most defined class of pyramid-associated domains was globally 3D-refined and further 3D-classified around the whole core region. The most defined class was globally 3D-refined and assigned as 3END-CORE. The most defined class of endo-associated domains was globally 3D-refined and further 3D-classified strictly around the endo domain. The most defined endo domain class was globally 3D-refined and assigned as 3END-ENDO. Final cryo-EM maps were refined and post-processed with their respective masks in RELION 3.1[8,9] and filtered by LocScale [10].

Supplementary Fig. 28

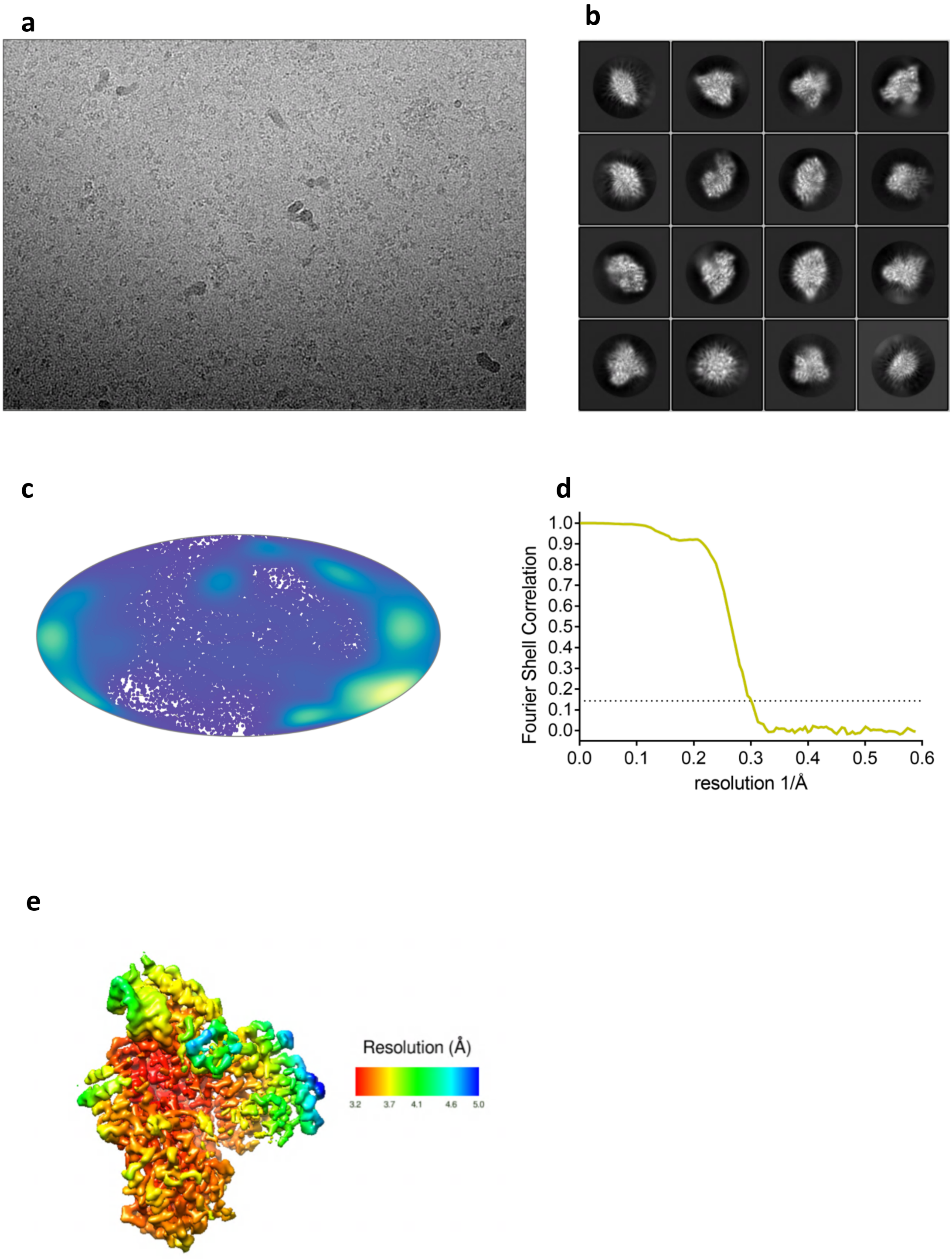

Supplementary Figure 28. Cryo-EM of LASV L pre-initiation complex

a, Representative micrograph of LASV L protein with 3' and 5' vRNAs in free standing ice after MotionCor2[6] correction at defocus of ~2.5 µm. **b,** 2D-class averages of the of LASV L protein with 3' and 5' vRNAs. **c,** Angular distribution for particle projections of PRE-INITIATION visualized on a globe-like plane. **d,** Fourier shell correlation (FSC) curves for PRE-INITIATION. The plot of the FSC between two independently refined half-maps shows the overall resolution of the two maps as indicated by the gold standard FSC 0.143 cut-off criteria[7]. **e,** Surface representation of local resolution distribution of PRE-INITIATION. The map is colored according to the local resolution calculated within the RELION software package[8,9]. Resolution is as indicated in the color bar.

Supplementary Fig. 29

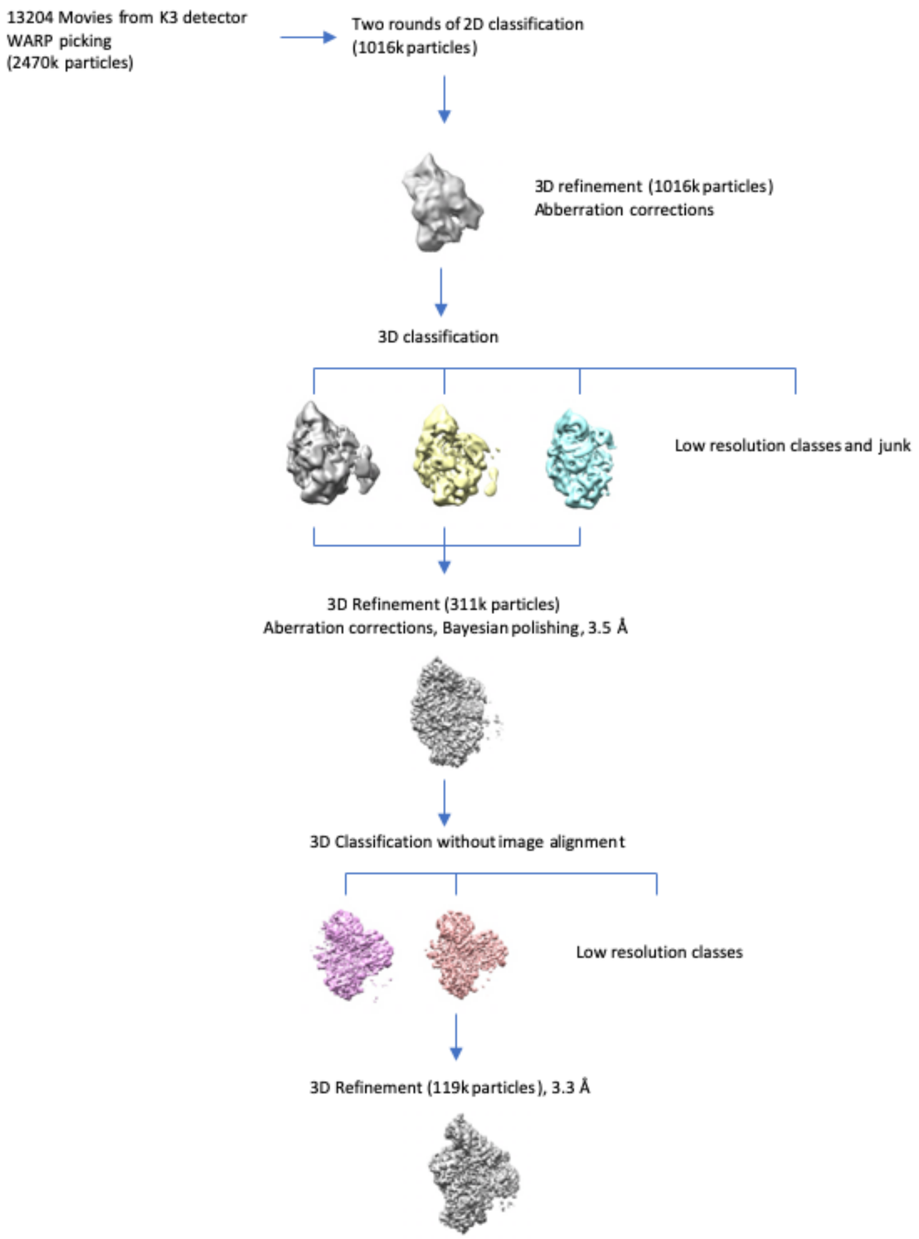

Supplementary Figure 29. Cryo-EM data 3D classification and refinement scheme of LASV L protein PRE-INITIATION complex

Summary of the cryo-EM 3D classification and refinement scheme of the PRE-INITIATION LASV L protein. CSSB DATA3 2D classes with well-defined secondary structure features were merged (1016k particles). The merged particles were classified into twelve 3D classes with angular assignment. Incomplete, low resolution, and damaged particle classes were excluded from further data analyses. Three most prominent 3D classes were merged globally 3D refined, aberration corrected and Bayesian polished [8,9] then further 3D classified. The best defined class was globally 3D-refined. Final cryo-EM map was refined and post-processed with their respective mask in RELION 3.1[8,9].

Supplementary Fig. 30

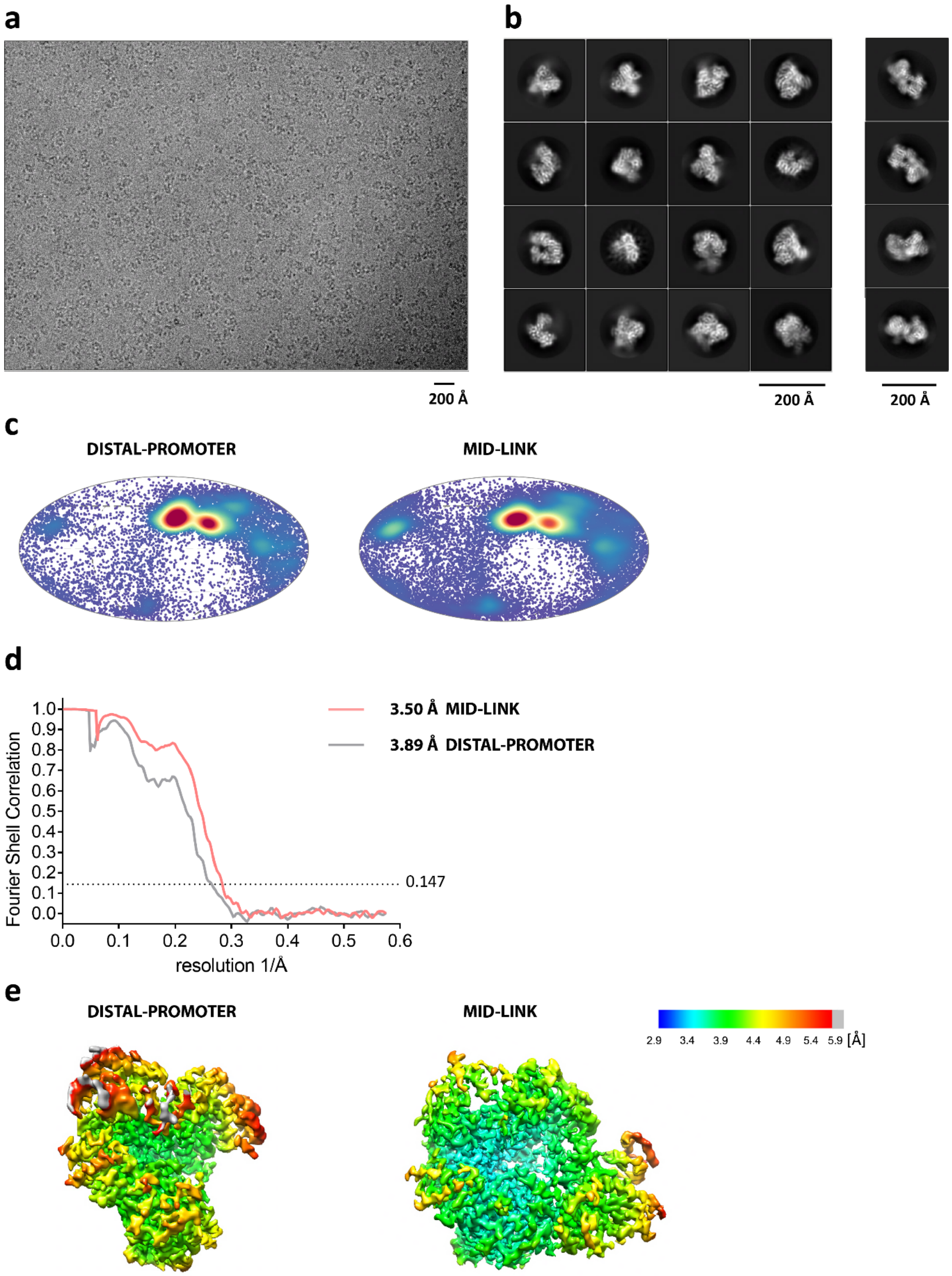

Supplementary Figure 30. Cryogenic electron microscopy of LASV L protein incubated with a truncated promoter

a, Representative micrograph of LASV L protein incubated with a truncated promoter in free standing ice after MotionCor2[6] correction at defocus of ~2.5 µm. **b,** 2D-class averages of the of LASV L protein incubated with a truncated promoter complex in monomeric (**left**) and dimeric (**right**) form. **c,** Angular distribution for particle projections of the DISTAL-PROMOTER, MID-LINK, respectively, visualized on a globe-like plane. **d,** Fourier shell correlation (FSC) curves for the DISTAL-PROMOTER (grey), MID-LINK (pink), respectively. The plot of the FSC between two independently refined half-maps shows the overall resolution of the two maps as indicated by the gold standard FSC 0.143 cut-off criteria[7]. **e,** Surface representation of local resolution distribution of the DISTAL-PROMOTER, MID-LINK, respectively. Maps are colored according to the local resolution calculated within the RELION software package[8,9]. Resolution is as indicated in the color bar.

Supplementary Fig. 31

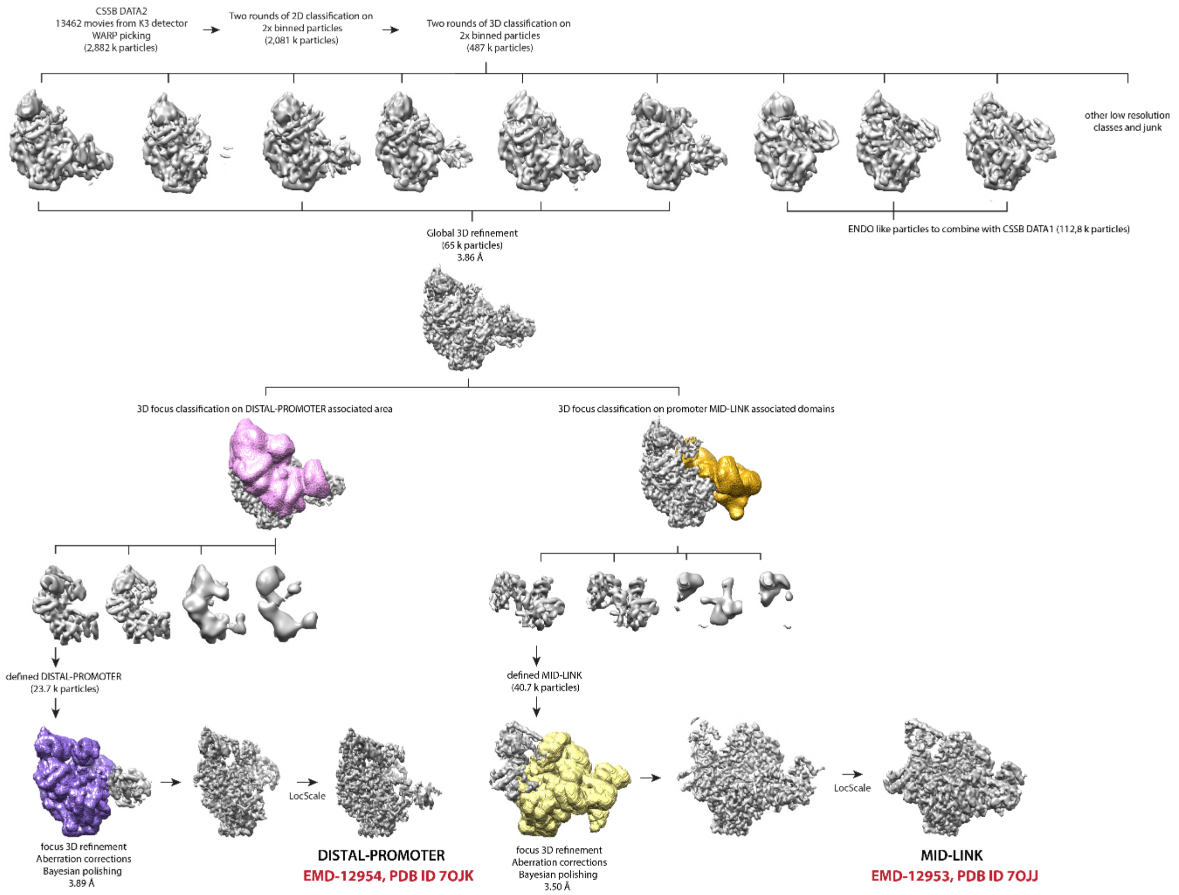

Supplementary Figure 31. Cryo-EM data 3D classification and refinement scheme LASV L protein incubated with a truncated promoter

Summary of the cryo-EM 3D classification and refinement scheme of the LASV L protein incubated with a truncated promoter. CSSB DATA2 2D classes with well-defined secondary structure features were merged (2,081k particles). The merged particles were classified into twelve 3D classes with angular assignment. Incomplete, low resolution, and damaged particle classes were excluded from further data analyses. Three classes, which possessed defined endo domain, were pooled with CSSB DATA1 (Figure EM2). Four classes, which possessed defined periphery around putative promoter-duplex binding site and mid-link domain, were merged and globally 3D-refined. The data were then in parallel focus 3D-classified around the DISTAL-PROMOTER regions and associated domains (left, pink) and MID-LINK-associated domains (right, orange). The most defined class of DISTAL-PROMOTER region was focused 3D-refined, excluding the MID-LINK region (purple). The most defined MID-LINK domain class was focus 3D-refined, excluding the DISTAL-PROMOTER region (yellow). Final cryo-EM maps were refined and post-processed with their respective masks in RELION 3.1[8,9] and filtered by LocScale1[10].

Supplementary Fig. 32

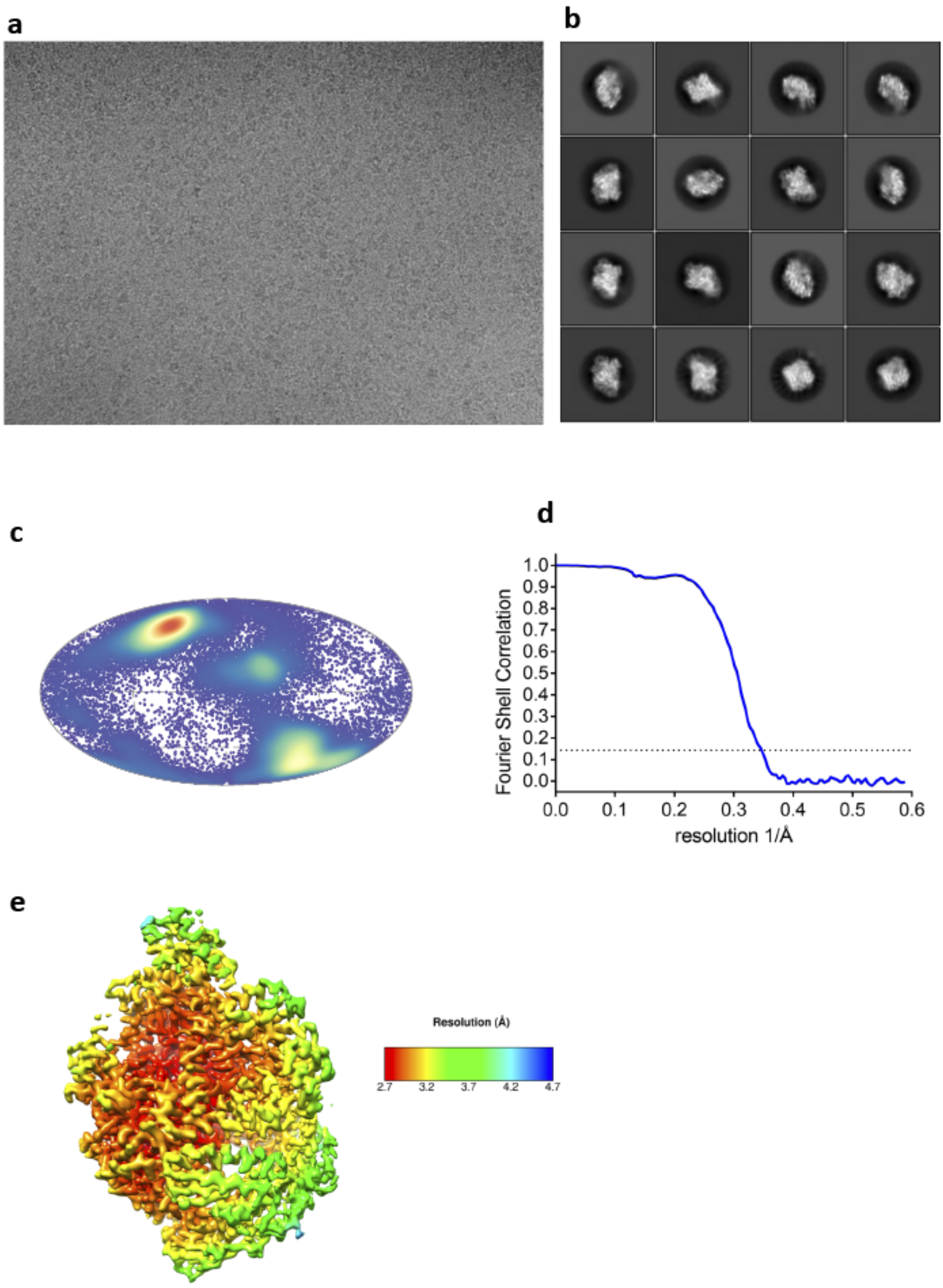

Supplementary Figure 32. Cryo-EM of LASV L protein ELONGATION complex

a, Representative micrograph of LASV L protein ELONGATION complex in free standing ice after MotionCor2[6] correction at defocus of ~2.0 µm. **b,** 2D-class averages of the of LASV L protein ELONGATION complex **c,** Angular distribution for particle projections of the ELONGATION complex visualized on a globe-like plane. **d,** Fourier shell correlation (FSC) curves for the ELONGATION. The plot of the FSC between two independently refined half maps shows the overall resolution of the two maps as indicated by the gold standard FSC 0.143 cut-off criteria[7]. **e,** Surface representation of local resolution distribution of the ELONGATION. Maps are colored according to the local resolution calculated within the RELION software package[8,9]. Resolution is as indicated in the color bar.

Supplementary Fig. 33

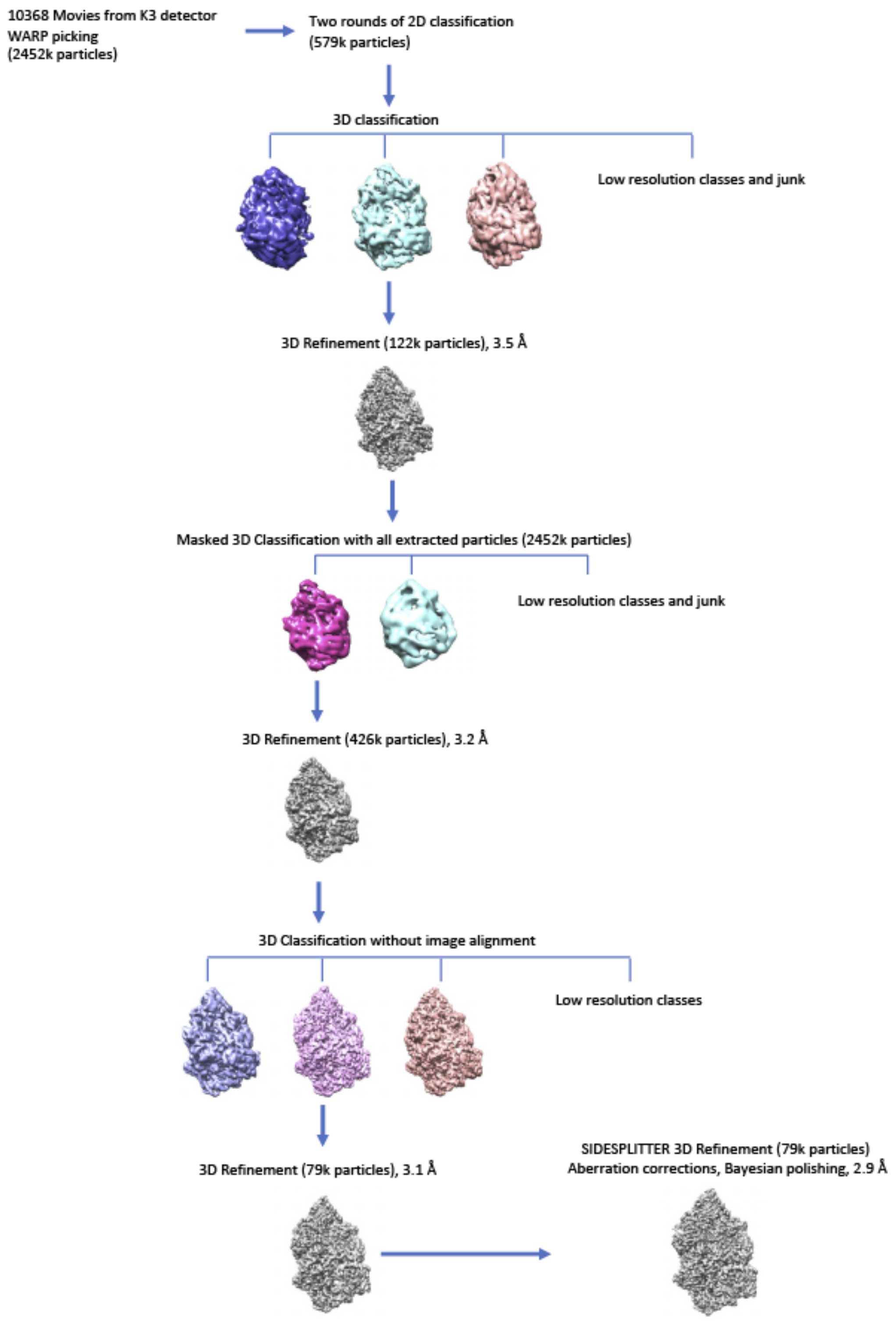

Supplementary Figure 33. Cryo-EM data 3D classification and refinement scheme of LASV L ELONGATION complex

Summary of the cryo-EM 3D classification and refinement scheme of the ELONGATION LASV L protein. CSSB DATA4 2D classes with well-defined secondary structure features were merged (579k particles). The merged particles were classified into ten 3D classes with angular assignment. The highest resolution class was 3D-Refined and then used as a reference for 3D classification with ten classes of all extracted particles (2452k). The most prominent 3D class was globally 3D-refined then further 3D classified then further 3D classified. The most defined class was globally 3D-refined. The final cryo-EM map was refined with SIDESPLITTER[11] and post-processed with their respective mask in RELION 3.1[8,9].

Supplementary Fig. 34

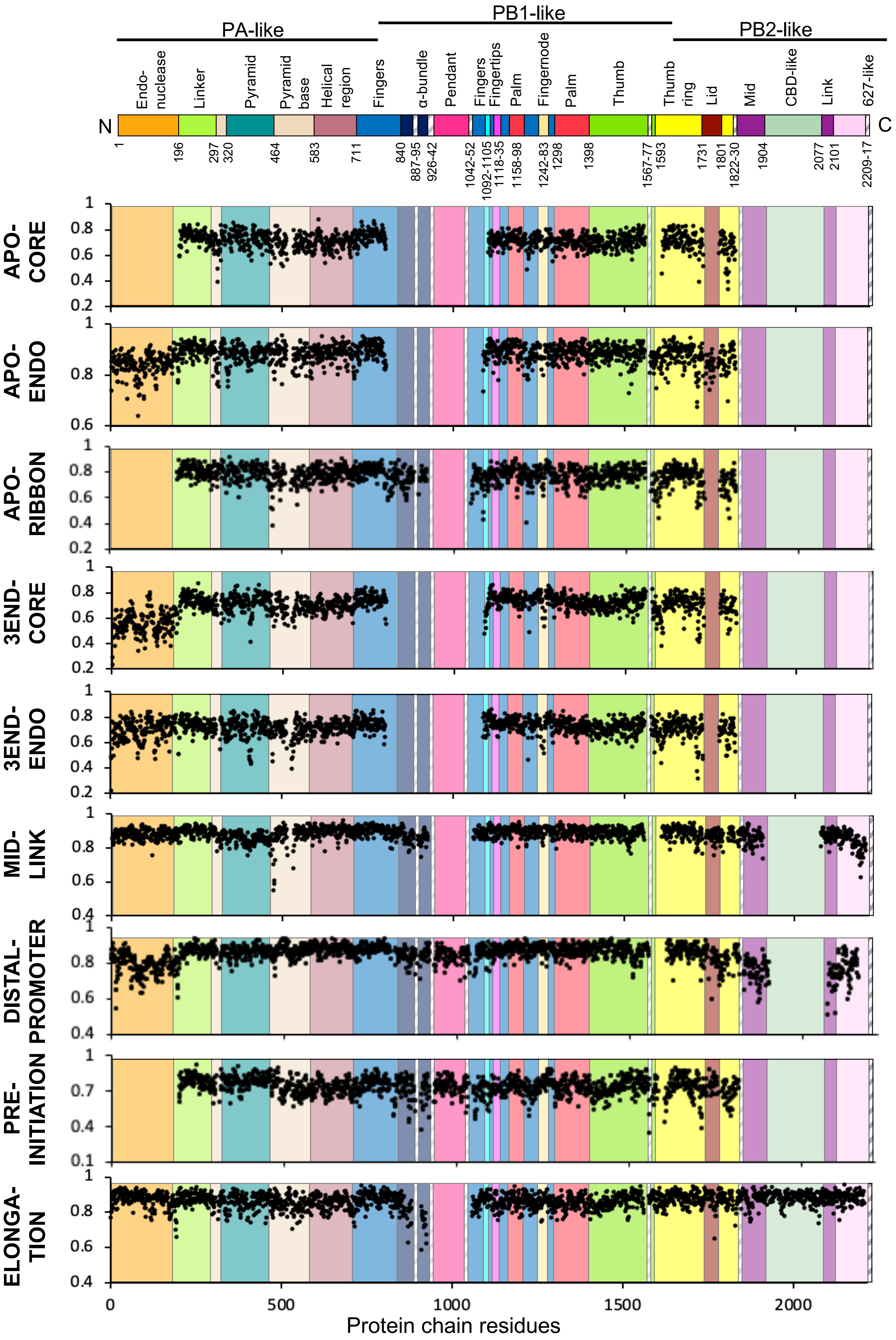

Supplementary Figure 34. Comparison of LASV L Protein Structures by TEMPy SMOC Score. Schematic representation of the domain structure of the LASV L protein as in Fig. 3 (top panel). For each LASV L protein structure, the SMOC scores per residue were calculated by TEMPy within CCP-EM [12,13] providing the respective resolution as described in Supplementary Table 2 and plotted revealing the quality of the local fit (bottom panels). The domain structure of LASV L protein as shown in the top panel has been added as background to each SMOC plot to highlight which domains are missing/present in each structure. Absolute numbers of SMOC scores are resolution-dependent, thus the y-axis scaling differs between the panels. For a detailed list of the residues included in each model please refer to Supplementary Table 3.

Supplementary References

1 Ashkenazy, H. *et al.* ConSurf 2016: an improved methodology to estimate and visualize evolutionary conservation in macromolecules. *Nucleic Acids Res* **44**, W344-350, doi:10.1093/nar/gkw408 (2016).

2 Lehmann, M. *et al.* Role of the C terminus of Lassa virus L protein in viral mRNA synthesis. *J Virol* **88**, 8713-8717, doi:10.1128/JVI.00652-14 (2014).

3 Wallace, A. C., Laskowski, R. A. & Thornton, J. M. LIGPLOT: a program to generate schematic diagrams of protein-ligand interactions. *Protein Eng* **8**, 127-134, doi:10.1093/protein/8.2.127 (1995).

4 Laskowski, R. A., Jablonska, J., Pravda, L., Varekova, R. S. & Thornton, J. M. PDBsum: Structural summaries of PDB entries. *Protein Sci* **27**, 129-134, doi:10.1002/pro.3289 (2018).

5 Xu, X. *et al.* Cryo-EM structures of Lassa and Machupo virus polymerases complexed with cognate regulatory Z proteins identify targets for antivirals. *Nat Microbiol* **6**, 921-931, doi:10.1038/s41564-021-00916-w (2021).

6 Zheng, S. Q. *et al.* MotionCor2: anisotropic correction of beam-induced motion for improved cryo-electron microscopy. *Nat Methods* **14**, 331-332, doi:10.1038/nmeth.4193 (2017).

7 Rosenthal, P. B. & Henderson, R. Optimal determination of particle orientation, absolute hand, and contrast loss in single-particle electron cryomicroscopy. *J Mol Biol* **333**, 721-745, doi:10.1016/j.jmb.2003.07.013 (2003).

8 Zivanov, J. *et al.* New tools for automated high-resolution cryo-EM structure determination in RELION-3. *Elife* **7**, doi:10.7554/eLife.42166 (2018).

9 Zivanov, J., Nakane, T. & Scheres, S. H. W. Estimation of high-order aberrations and anisotropic magnification from cryo-EM data sets in RELION-3.1. *IUCrJ* **7**, 253-267, doi:10.1107/S2052252520000081 (2020).

10 Jakobi, A. J., Wilmanns, M. & Sachse, C. Model-based local density sharpening of cryo-EM maps. *Elife* **6**, doi:10.7554/eLife.27131 (2017).

11 Ramlaul, K., Palmer, C. M., Nakane, T. & Aylett, C. H. S. Mitigating local over-fitting during single particle reconstruction with SIDESPLITTER. *J Struct Biol* **211**, 107545, doi:10.1016/j.jsb.2020.107545 (2020).

12 Cragnolini, T. *et al.* TEMPy2: a Python library with improved 3D electron microscopy density-fitting and validation workflows. *Acta Crystallogr D Struct Biol* **77**, 41-47, doi:10.1107/S2059798320014928 (2021).

13 Burnley, T., Palmer, C. M. & Winn, M. Recent developments in the CCP-EM software suite. *Acta Crystallogr D Struct Biol* **73**, 469-477, doi:10.1107/S2059798317007859 (2017).
