# Supplementary material for: Conformational changes in Lassa virus L protein associated with promoter binding and RNA synthesis activity
Source: Nat Commun. 2021 Dec 2;12:7018. doi: 10.1038/s41467-021-27305-5 (PMC8639829; doi:10.1038/s41467-021-27305-5)
Supplement: Supplementary file 3 — Description of Additional Supplementary Files [file 41467_2021_27305_MOESM3_ESM.pdf]

Description of Additional Supplementary Files

File Name: Supplementary Data 1
Description: Alignment of full-length L protein sequences of Old World and New World arenaviruses. L protein sequences (virus abbreviations, GenBank IDs listed) were aligned using ClustalOmega (https://www.ebi.ac.uk/Tools/msa/clustal o/) [1] with manual adjustments. The alignment was presented by ESPript [2] with secondary structure information given for LASV Bantou 289 L protein (PDB 7OJN). Regions of the L protein are labelled and marked with different colors according to Fig. 3. Conserved RdRp motifs are labelled according to Fig. 4e. All numbers given refer to LASV Bantou 289 L protein sequence.

1. Madeira F, Park YM, Lee J, Buso N, Gur T, Madhusoodanan N, Basutkar P, Tivey ARN, Potter SC, Finn RD, Lopez R. The EMBL-EBI search and sequence analysis tools APIs in 2019. Nucleic Acids Res. 2019;47(W1):W636-W41.
2. Robert X, Gouet P. Deciphering key features in protein structures with the new ENDscript server. Nucleic Acids Res. 2014;42(Web Server issue):W320-4.

File Name: Supplementary Movie 1
Description: The PRE- INITIATION structure. This movie highlights the interactions between the L protein and the 3' and 5' promoter RNA. Hydrogen bonds and electrostatic interactions are shown as dotted black lines.

File Name: Supplementary Movie 2
Description: The ELONGATION structure. This movie presents the L protein stalled in an early elongation state with a non-hydrolysable UTP (UMPNPP) in the RdRp active site. Hydrogen bonds and electrostatic interactions are shown as dotted black lines. The movie further shows the putative product and template exit channels highlighting an interaction of the thumb domain with the RNA potentially involved in strand separation of template and product RNA duplex.

File Name: Supplementary Movie 3
Description: Overview of the L protein structure. This movie introduces the different domains of the LASV L protein (ELONGATION structure).
