# Supplementary material for: Conformational changes in Lassa virus L protein associated with promoter binding and RNA synthesis activity
Source: Nat Commun. 2021 Dec 2;12:7018. doi: 10.1038/s41467-021-27305-5 (PMC8639829; doi:10.1038/s41467-021-27305-5)
Supplement: Supplementary file 4 — Supplementary Data 1 [file 41467_2021_27305_MOESM4_ESM.pdf]

Supplementary Data 1. Alignment of full-length L protein sequences of Old World and New World arenaviruses

Conformational changes in Lassa virus L protein associated with promoter binding and RNA synthesis activity

Tomas Kouba[1], Dominik Vogel[2], Sigurdur R. Thorkelsson[3], Emmanuelle R. J. Quemin[3], Harry M. Williams[2], Morlin Milewski[2], Carola Busch[2], Stephan Günther[2], Kay Grünewald[3], Maria Rosenthal[2,*] and Stephen Cusack[1,*]

1- European Molecular Biology Laboratory, Grenoble, France

2- Bernhard Nocht Institute for Tropical Medicine, Hamburg, Germany

3- Centre for Structural Systems Biology, Leibniz Institute for Experimental Virology, University of Hamburg, Hamburg, Germany

These authors contributed equally: Tomas Kouba, Dominik Vogel, Sigurdur R. Thorkelsson

These authors jointly supervised this work: Maria Rosenthal and Stephen Cusack

* To whom correspondence should be addressed: rosenthal@bnitm.de and cusack@embl.fr

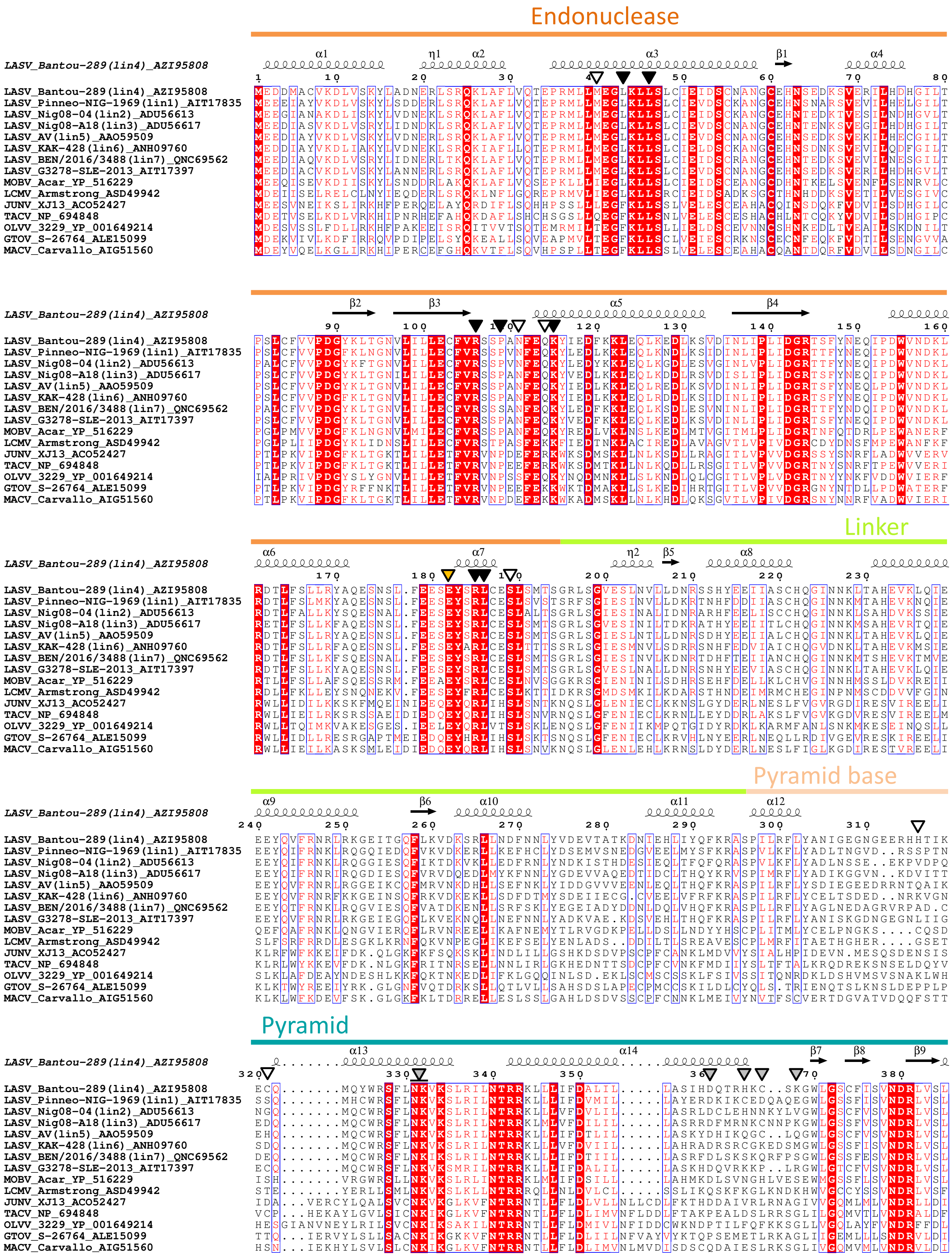

▼ – general defect in L protein activity upon mutation observed in Lassa virus mini-replicon system

▼ – selective transcriptional defect of L protein upon mutation observed in Lassa virus mini-replicon system

▽ – no or weak effect on L protein activity upon mutation observed in Lassa virus mini-replicon system

▽ – intermediate effect on L protein activity upon mutation observed in Lassa virus mini-replicon system

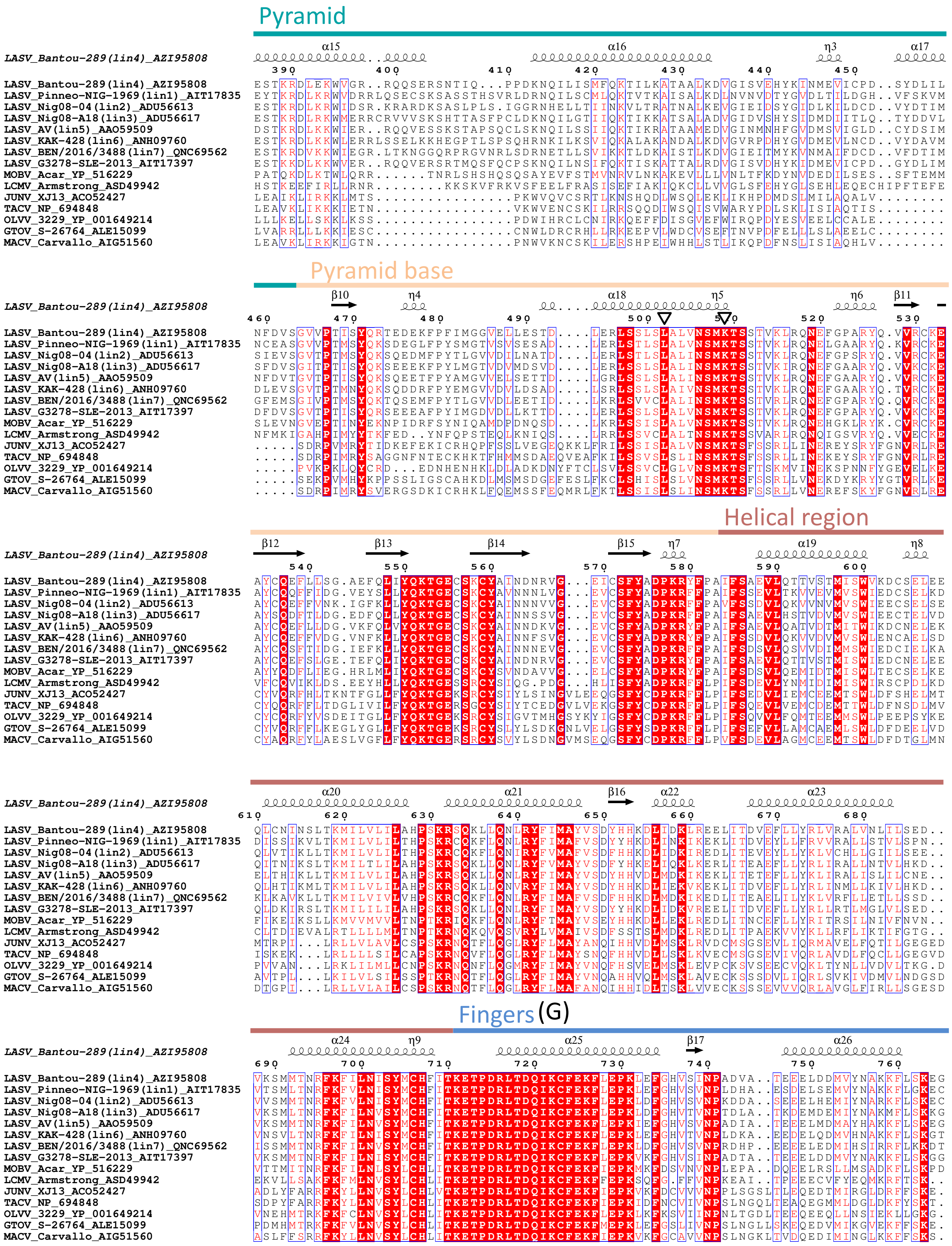

▼ – general defect in L protein activity upon mutation observed in Lassa virus mini-replicon system

▼ – selective transcriptional defect of L protein upon mutation observed in Lassa virus mini-replicon system

▽ – no or weak effect on L protein activity upon mutation observed in Lassa virus mini-replicon system

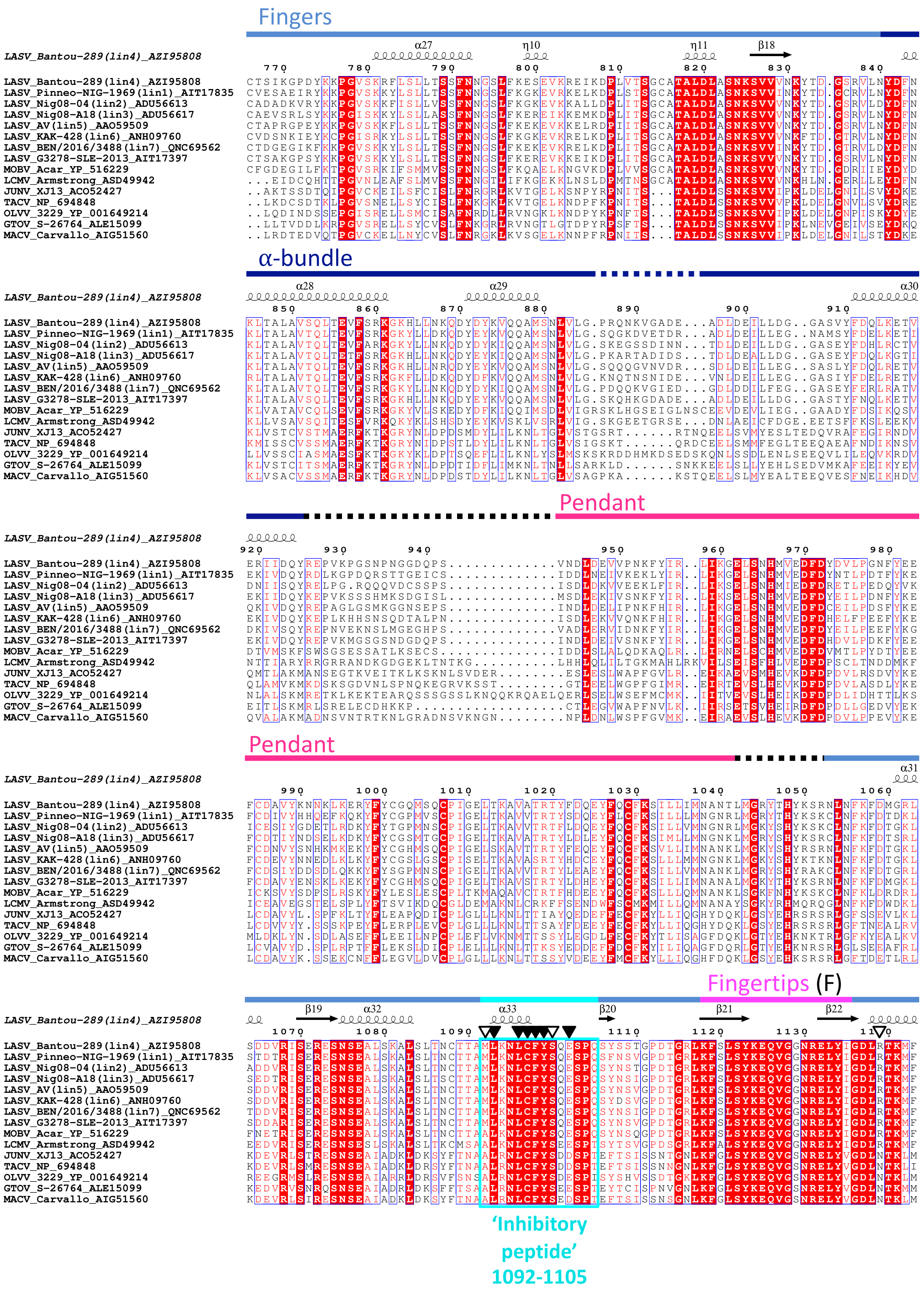

▼ – general defect in L protein activity upon mutation observed in Lassa virus mini-replicon system

▼ – selective transcriptional defect of L protein upon mutation observed in Lassa virus mini-replicon system

▽ – no or weak effect on L protein activity upon mutation observed in Lassa virus mini-replicon system

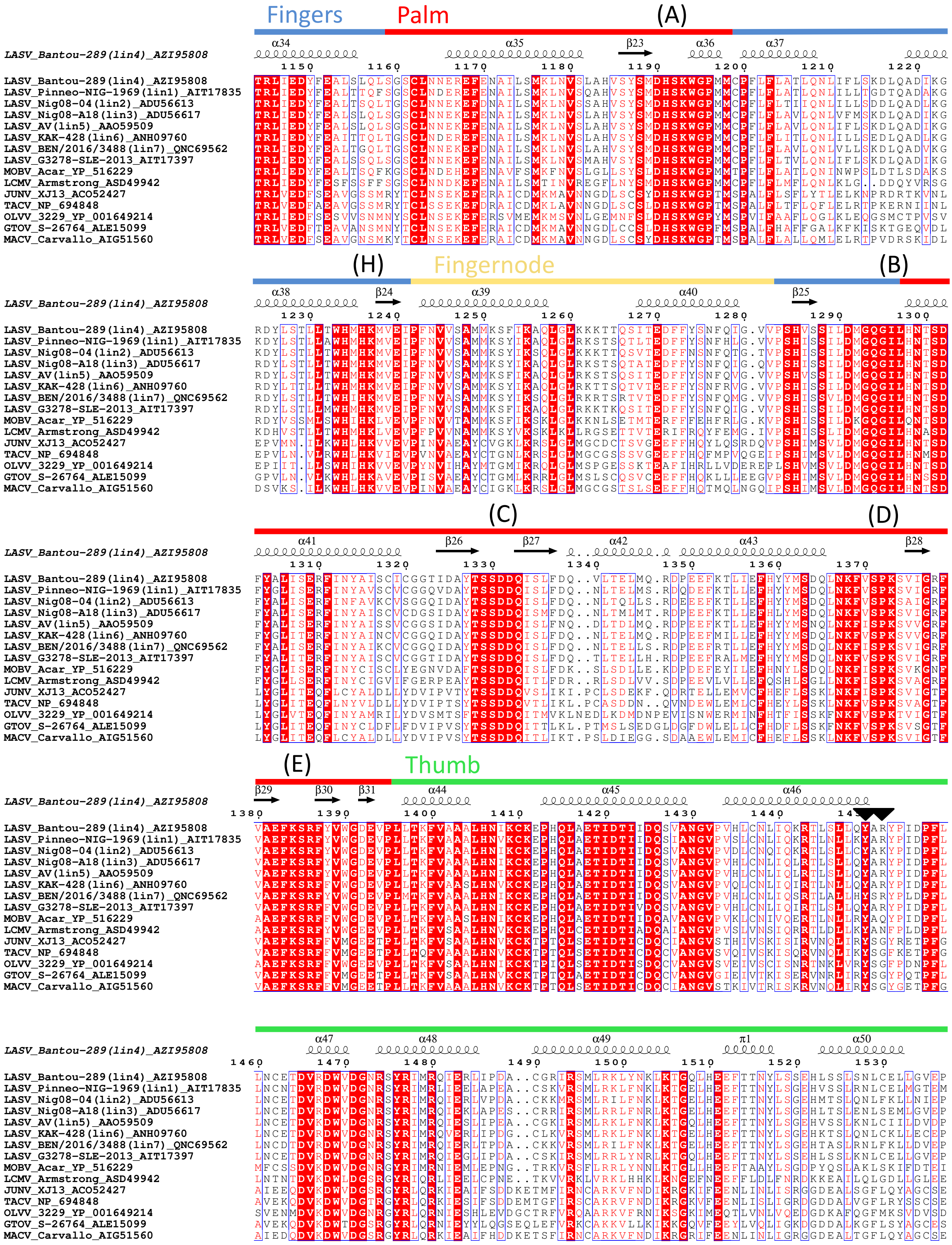

▼ – general defect in L protein activity upon mutation observed in Lassa virus mini-replicon system

▼ – selective transcriptional defect of L protein upon mutation observed in Lassa virus mini-replicon system

▽ – no or weak effect on L protein activity upon mutation observed in Lassa virus mini-replicon system

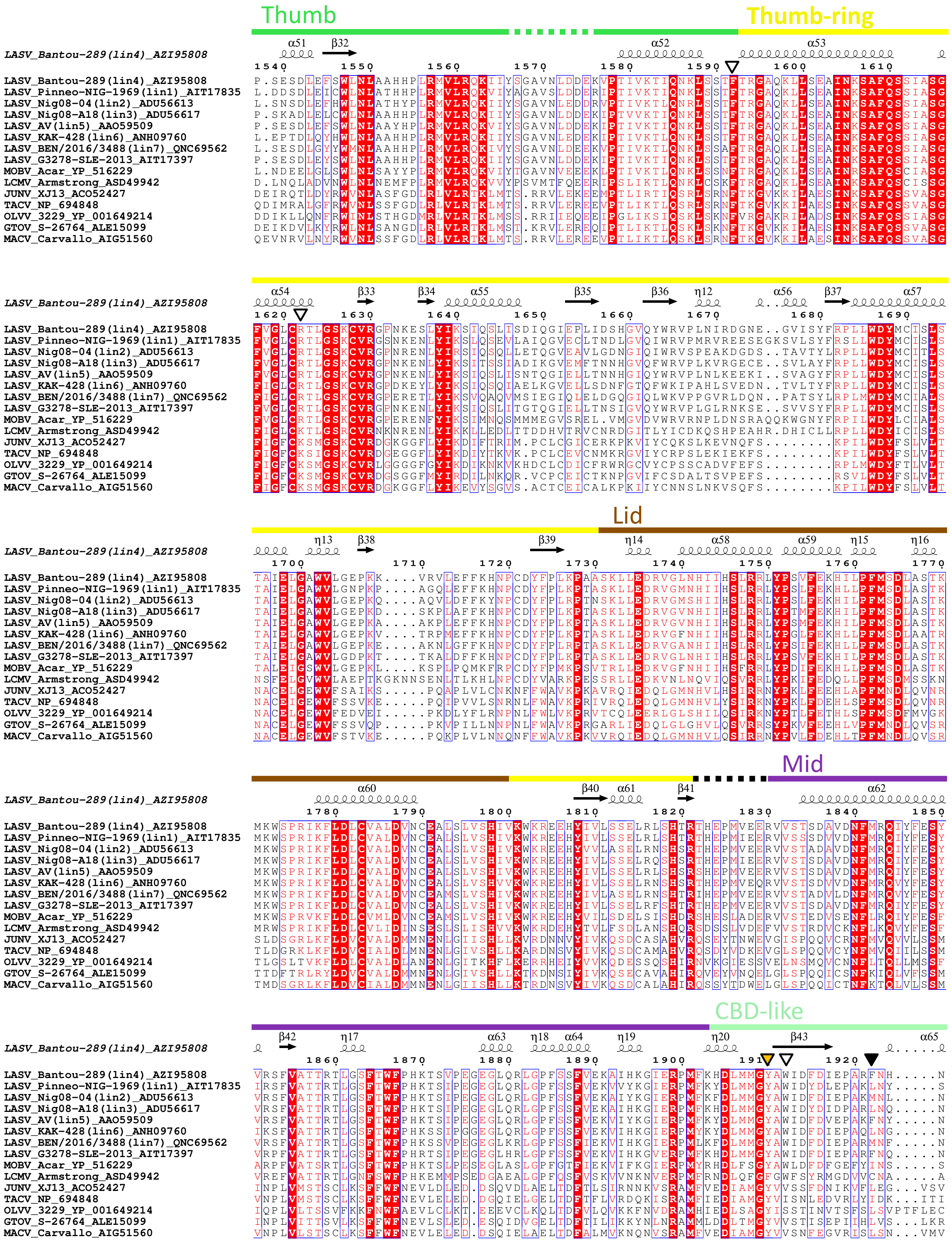

▼ – general defect in L protein activity upon mutation observed in Lassa virus mini-replicon system

▼ (orange) – selective transcriptional defect of L protein upon mutation observed in Lassa virus mini-replicon system

▽ – no or weak effect on L protein activity upon mutation observed in Lassa virus mini-replicon system

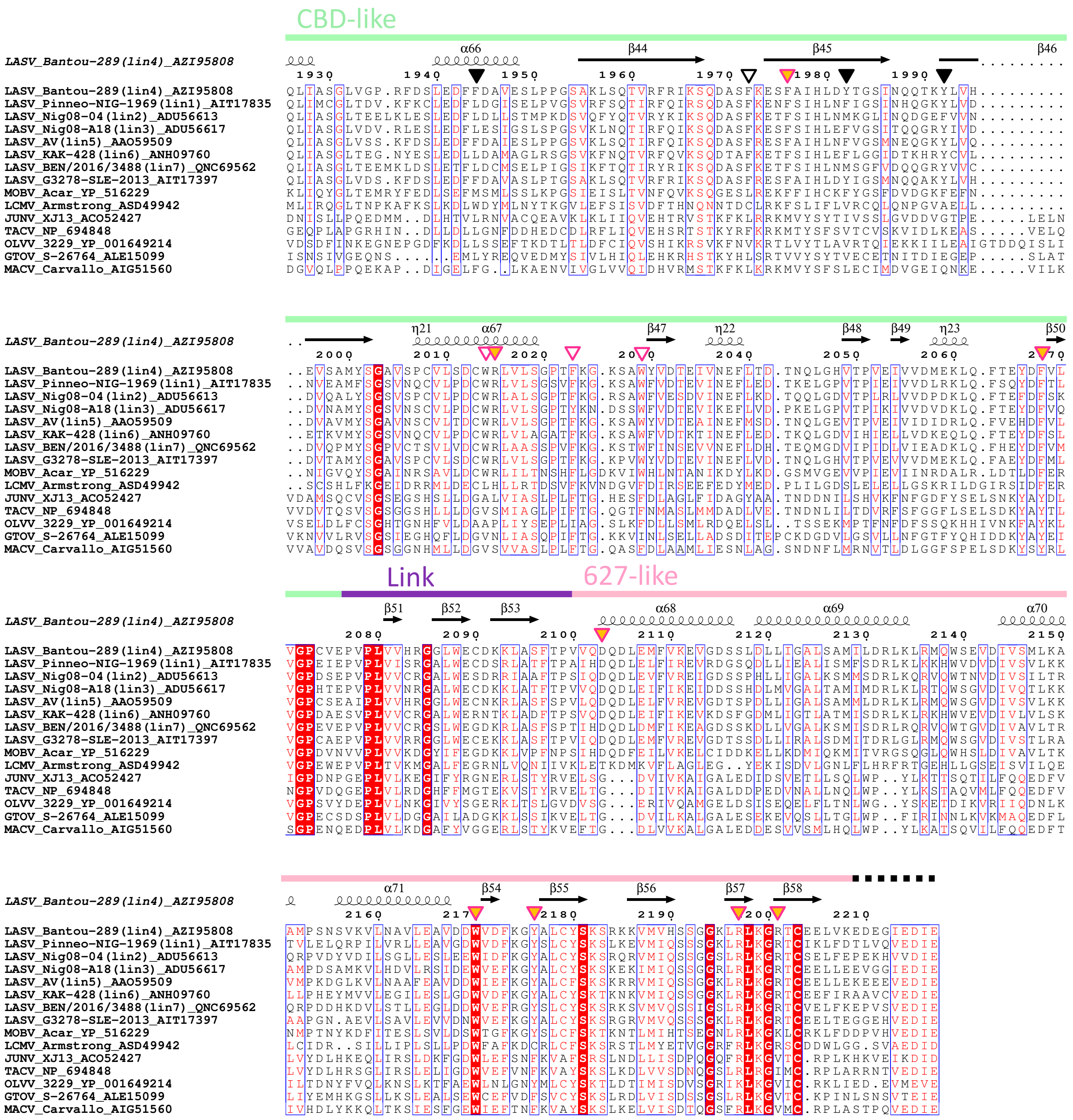

▽ – selective transcriptional defect of L protein upon mutation observed in Lassa virus mini-replicon system (Lehmann et al. 2014)

▽ – no or weak effect on L protein activity upon mutation observed in Lassa virus mini-replicon system (Lehmann et al. 2014)

▼ – general defect in L protein activity upon mutation observed in Lassa virus mini-replicon system

▽ – selective transcriptional defect of L protein upon mutation observed in Lassa virus mini-replicon system

▽ – no or weak effect on L protein activity upon mutation observed in Lassa virus mini-replicon system

▽ – intermediate effect on L protein activity upon mutation observed in Lassa virus mini-replicon system
