# Supplementary material for: Conformational changes in Lassa virus L protein associated with promoter binding and RNA synthesis activity
Source: Nat Commun. 2021 Dec 2;12:7018. doi: 10.1038/s41467-021-27305-5 (PMC8639829; doi:10.1038/s41467-021-27305-5)
Supplement: Supplementary file 8 — Reporting Summary [file 41467_2021_27305_MOESM8_ESM.pdf]

nature portfolio

Corresponding author(s): Stephen Cusack, Maria Rosenthal

Last updated by author(s): Oct 20, 2021

Reporting Summary

Nature Portfolio wishes to improve the reproducibility of the work that we publish. This form provides structure for consistency and transparency in reporting. For further information on Nature Portfolio policies, see our Editorial Policies and the Editorial Policy Checklist.

Statistics

For all statistical analyses, confirm that the following items are present in the figure legend, table legend, main text, or Methods section.

n/a	Confirmed	
☐	☒	The exact sample size (*n*) for each experimental group/condition, given as a discrete number and unit of measurement
☐	☒	A statement on whether measurements were taken from distinct samples or whether the same sample was measured repeatedly
☒	☐	The statistical test(s) used AND whether they are one- or two-sided<br>*Only common tests should be described solely by name; describe more complex techniques in the Methods section.*
☒	☐	A description of all covariates tested
☒	☐	A description of any assumptions or corrections, such as tests of normality and adjustment for multiple comparisons
☐	☒	A full description of the statistical parameters including central tendency (e.g. means) or other basic estimates (e.g. regression coefficient) AND variation (e.g. standard deviation) or associated estimates of uncertainty (e.g. confidence intervals)
☒	☐	For null hypothesis testing, the test statistic (e.g. *F*, *t*, *r*) with confidence intervals, effect sizes, degrees of freedom and *P* value noted<br>*Give P values as exact values whenever suitable.*
☒	☐	For Bayesian analysis, information on the choice of priors and Markov chain Monte Carlo settings
☒	☐	For hierarchical and complex designs, identification of the appropriate level for tests and full reporting of outcomes
☒	☐	Estimates of effect sizes (e.g. Cohen's *d*, Pearson's *r*), indicating how they were calculated

Our web collection on statistics for biologists contains articles on many of the points above.

Software and code

Policy information about availability of computer code

Data collection	Cryo-EM data were acquired using EPU software 2.8 (FEI); fluorescence signals in gels were detected with the VILBER LOURMAT FUSION SL4 software Fusion Capt V16.07; phosphor screen autoradiography detection was done using Fujifilm Typhoon FLA-7000 software version 1.3 Build 1.3.0.105
Data analysis	Single particle cryo-EM: multi-frame cryo-EM micrographs were motion corrected with the Relion 3.1 implementation of MotionCor2; contrast-transfer function parameter calculation was done with CTFFIND 4.1; astigmatism correction was done with CTFrefine within RELION 3.1; particles were selected with WARP 1.0.9; further 2D and 3D cryo-EM image processing was performed in RELION 3.1; for one dataset SIDESPLITTER 1.0 was used for 3D refinement; local resolution was calculated using RELION 3.1 and reference-based local amplitude scaling was performed by LocScale within the CCP-EM software suite v1.5.<br>Model-building was performed with Coot (versions 0.8.9.2 and 0.8.9.0) and real-space refinement with Phenix (versions 1.19.2-4158; 1.19.0-4092, and 1.19.1-4122); secondary structure prediction was done using JPRED4 (https://www.compbio.dundee.ac.uk/jpred/); 3D structure prediction was done using AlphaFold2 (https://colab.research.google.com/github/sokrypton/ColabFold/blob/main/AlphaFold2.ipynb); sequence alignments were performed using ClustalOmega (https://www.ebi.ac.uk/Tools/msa/clustalo/) and presented using ESPript 3.0 (https://espript.ibcp.fr/ESPript/cgi-bin/ESPript.cgi); buried surface areas were determined using the Protein interfaces, surfaces and assemblies' service PISA at the European Bioinformatics Institute (https://www.ebi.ac.uk/pdbe/pisa/); Segment based Manders' Overlap Coefficient (SMOC) scores were calculated in TEMPy within CCP-EM 1.5.0; structure presentation was done using PyMOL (Schrödinger, versions 2.3.4 and 2.5.0) and UCSF ChimeraX 1.0; analysis of conservation on the structure level based on an alignment was done using the ConSurf web server (https://consurf.tau.ac.il/); surface electrostatics were calculated by APBS electrostatics plugin within PyMOL 2.5.0 (Schrödinger); mini-replicon data analysis was done in Prism 7.0d; Northern blot quantification was done in ImageJ 2.0.0-rc-43/1.50e

For manuscripts utilizing custom algorithms or software that are central to the research but not yet described in published literature, software must be made available to editors and reviewers. We strongly encourage code deposition in a community repository (e.g. GitHub). See the Nature Portfolio guidelines for submitting code & software for further information.

Data

Policy information about availability of data

All manuscripts must include a data availability statement. This statement should provide the following information, where applicable:

- Accession codes, unique identifiers, or web links for publicly available datasets
- A description of any restrictions on data availability
- For clinical datasets or third party data, please ensure that the statement adheres to our policy

Data and unique biological materials are available from the corresponding authors. Coordinates and structure factors or maps generated in this study have been deposited in the wwwPDB or EMDB:
Apo-structure of Lassa virus L protein (well-resolved polymerase core) [APO-CORE] EMD-12807 [https://www.ebi.ac.uk/pdbe/entry/emdb/EMD-12807] (Fo-wing), PDB ID 7OCH [http://doi.org/10.2210/pdb7OCH/pdb]
Apo-structure of Lassa virus L protein (well-resolved endonuclease) [APO-ENDO] EMD-12860 [https://www.ebi.ac.uk/pdbe/entry/emdb/EMD-12860] (Fo-wing), PDB ID 7OE3[http://doi.org/10.2210/pdb7OE3/pdb]
Apo-structure of Lassa virus L protein (well-resolved alpha-ribbon) [APO-RIBBON] EMD-12953 [https://www.ebi.ac.uk/pdbe/entry/emdb/EMD-12953] (Fo-wing), PDB ID 7OE7[http://doi.org/10.2210/pdb7OE7/pdb]
Lassa virus L protein bound to 3' promoter RNA (well-resolved polymerase core and 3' RNA secondary binding site) [3END-CORE] EMD-12862 [https://www.ebi.ac.uk/pdbe/entry/emdb/EMD-12862] (Fo-wing), PDB ID 7OEA [http://doi.org/10.2210/pdb7OEA/pdb]
Lassa virus L protein bound to 3' promoter RNA (well-resolved endonuclease) [3END-ENDO] EMD-12863 [https://www.ebi.ac.uk/pdbe/entry/emdb/EMD-12863] (Fo-wing), PDB ID 7OEB [http://doi.org/10.2210/pdb7OEB/pdb]
Lassa virus L protein in a pre-initiation conformation [PRE-INITIATION] EMD-12955 [https://www.ebi.ac.uk/pdbe/entry/emdb/EMD-12955] (Fo-wing), PDB ID 7OJL [http://doi.org/10.2210/pdb7OJL/pdb]
Lassa virus L protein with endonuclease and C-terminal domains in close proximity [MID-LINK] EMD-12861 [https://www.ebi.ac.uk/pdbe/entry/emdb/EMD-12861] (Fo-wing), PDB ID 7OJJ [https://www.rcsb.org/structure/7OJJ]
Lassa virus L protein bound to the distal promoter duplex [DISTAL-PROMOTER] EMD-12954 [https://www.ebi.ac.uk/pdbe/entry/emdb/EMD-12954] (Fo-wing), PDB ID 7OJK [http://doi.org/10.2210/pdb7OJK/pdb]
Lassa virus L protein in an elongation conformation [ELONGATION] EMD-12956 [https://www.ebi.ac.uk/pdbe/entry/emdb/EMD-12956] (Fo-wing), PDB ID 7OJN [http://doi.org/10.2210/pdb7OJN/pdb]
Supplementary data and source data are provided with this paper.

Field-specific reporting

Please select the one below that is the best fit for your research. If you are not sure, read the appropriate sections before making your selection.

☒ Life sciences ☐ Behavioural & social sciences ☐ Ecological, evolutionary & environmental sciences

For a reference copy of the document with all sections, see nature.com/documents/nr-reporting-summary-flat.pdf

Life sciences study design

All studies must disclose on these points even when the disclosure is negative.

Sample size	For mini-replicon experiments, at least 3 biological replicates were performed to allow calculation of mean and SD.
Data exclusions	Data were only excluded if the experiment technically failed, i.e. controls did not work.
Replication	For mini-replicon experiments, at least 3 biological replicates were performed to ensure reproducibility, all replicates were successful when the respective controls were ok.
Randomization	not applicable, there are no clinical data included in this manuscript
Blinding	not applicable, there are no clinical data included in this manuscript

Reporting for specific materials, systems and methods

We require information from authors about some types of materials, experimental systems and methods used in many studies. Here, indicate whether each material, system or method listed is relevant to your study. If you are not sure if a list item applies to your research, read the appropriate section before selecting a response.

Materials & experimental systems

n/a	Involved in the study	
☐	☒	Antibodies
☐	☒	Eukaryotic cell lines
☒	☐	Palaeontology and archaeology
☒	☐	Animals and other organisms
☒	☐	Human research participants
☒	☐	Clinical data
☒	☐	Dual use research of concern

Methods

n/a	Involved in the study	
☒	☐	ChIP-seq
☒	☐	Flow cytometry
☒	☐	MRI-based neuroimaging

Antibodies

Antibodies used	mouse monoclonal anti-FLAG M2 antibody, peroxidase-conjugated (A8592; Sigma-Aldrich), dilution 1:10,000 used
Validation	validated by manufacturer for one-step detection by immunocytochemistry, ELISA, or Western blotting, recognizes epitope sequence DYKDDDDK, peroxidase conjugate (covalent), additionally we used mock transfected cells as negative controls on every Western blot

Eukaryotic cell lines

Policy information about cell lines

Cell line source(s)	BSR-T7 cell line was obtained from K.K. Conzelmann (Buchholz et al. J Virol 1999 doi: 10.1128/JVI.73.1.251-259.1999), HighFive insect cells were purchased from Thermo Fisher Scientific (catalog number B85502)
Authentication	BSR-T7 authentication was done by STR analysis (last done in 2013), BSR-T7 cells are regularly treated with Geneticin for selection of T7 expressing cells, HighFive cells were not authenticated as they were commercially available and only used for expression and not to test any scientific hypotheses
Mycoplasma contamination	cell lines are regularly tested for mycoplasma contamination and only kept if negative
Commonly misidentified lines (See ICLAC register)	no commonly misidentified cell lines were used in this study, BSR-T7 cells are under constant selective pressure by Geniticin
